# A Microfluidic Digital Shutter of Liquid–Liquid Interface for Fabrication of Multifaceted Hydrogel Microfiber Structure

**DOI:** 10.1002/advs.202510989

**Published:** 2025-12-29

**Authors:** Dongrui Zhang, Jiangyue Liu, Hao Ye, Yu Shen, Haoran Su, Zhuqing Liang, Kexin Li, Xing Zhang, Shiyi Yang, Yunfei Lu, Zhexi Zhang, Sihang Liu, Yi Huang, Xiao Liu, Yubo Fan

**Affiliations:** ^1^ Key Laboratory of Biomechanics and Mechanobiology (Beihang University) Ministry of Education Beijing Advanced Innovation Center for Biomedical Engineering School of Biological Science and Medical Engineering Beihang University Beijing 100083 China; ^2^ Hangzhou International Innovation Institute Beihang University Hangzhou 311115 China; ^3^ National Key Laboratory of Science and Technology on Aero‐Engine Aero‐Thermodynamics Research Institute of Aero‐Engine Beihang University Beijing 100191 China; ^4^ School of Engineering Medicine Beihang University Beijing 100083 China

**Keywords:** hydrogel microfiber, liquid–liquid interface, microfluidic chip

## Abstract

The digital logic elements based on microfluidic devices are proposed for complex fluidic control. However, there is no logic microfluidic device for fabrication. Moreover, the precise fabrication of anisotropic hydrogel microparticles with programmable geometries has long remained a central challenge in soft matter physics, materials science, and biomedical engineering. Here, a programmable passive digital shutter mechanism based on multiphase liquid–liquid interface dynamics is introduced for the precise fabrication of hydrogel microfibers in a microfluidic platform. There are three fluids in the devices, where two immiscible phases are designed for creating a digital shutter and two aqueous phases for hydrogel formation. It is found that the liquid bridge of hydrogel solution at the liquid–liquid interface has a critical stable length, and the digital shutter is achieved by toggling between liquid bridge maintenance and breakup. By modulating the digital shutter, deterministic control over geometry in terms of length, aspect ratio, curvature, and torsion, and produce microfibers in diverse shapes, including linear, helical, tadpole‐like, and spherical is achieved. To highlight its potential for tissue engineering, active materials, and soft microrobotics, the platform is utilized to generate cell‐laden microfibers with high resolution and viability, and magnetically responsive microfibers.

## Graphical abstract

A programmable passive digital shutter based on multiphase liquid–liquid interface dynamics is introduced for precise fabrication of hydrogel microfibers. By modulating the shutter operation, microfibers with tunable geometry—including linear, helical, tadpole‐like, and spherical forms—are produced. The platform enables the creation of cell‐laden and magnetically responsive microfibers for advanced biofabrication and soft microrobotics.

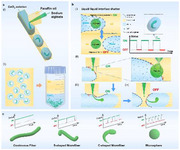

## 1 Introduction

Anisotropic microparticles with defined morphology, such as curvature and composite composition, can break through the limitations of general spherical particles and find wide applications in sensing,^[^ ^1^, ^2^ ^]^ drug delivery, cell therapy,^[^ ^3^, ^4^ ^]^ collective phase transformation,^[^ ^5^ ^]^ colloidal robotics,^[^ ^6^ ^]^ tissue engineering.^[^ ^7^ ^]^ In the human body, anisotropic fiber‐shaped repeating units are made up of several basic tissues, including the muscle, vasculature, and nervous tissue. Therefore, fiber‐shaped hydrogel microparticles encapsulating cells can be used as building block units for reproducing complex structures of natural organs in vitro.^[^ ^8^, ^9^, ^10^ ^]^ Furthermore, unlike spherical particles, the geometry of fiber‐shaped microparticles affects a range of properties from injectability^[^ ^9^ ^]^ to assembly and cell behavior.^[^ ^11^, ^12^ ^]^

Microfluidics offers a powerful platform for anisotropic microparticle generation, enabling precise and independent control over morphology, size, and monodispersity, compared to techniques like molding,^[^ ^12^ ^]^ emulsions,^[^ ^13^ ^]^ photolithography,^[^ ^14^ ^]^ and mechanical fragmentation.^[^ ^9^ ^]^ Typically, when one liquid flows into another immiscible liquid in microfluidic devices such as T‐junction,^[^ ^15^ ^]^ flow focusing,^[^ ^16^ ^]^ or co‐flow,^[^ ^17^, ^18^ ^]^ hydrodynamic instabilities driven by surface tension and shear stress lead to drop breakup.^[^ ^19^, ^20^ ^]^ As a result, microgels formed in these systems are generally limited to spherical geometries.^[^^]^ Combining microfluidics with lithography enables the fabrication of morphologically complex microgels,^[^ ^11^, ^21^, ^22^ ^]^ but the harsh processing conditions and limited biocompatible materials compromise cell viability. Thus, generating fiber‐shaped microparticles with controllable geometry under biocompatible conditions and in a high‐throughput manner remains challenging.

Here, we introduce a dynamic interface between two immiscible liquids in a microfluidics system as a digital shutter, which programmatically switches between two states during the maintenance and breakup of a liquid bridge for generating multifaceted hydrogel microfibers. The microfluidics system is the combination of a canonical T‐junction and co‐flow devices, where three‐fluid systems make up two immiscible fluid systems for digital shutter by capillary instability and one aqueous two‐phase system for hydrogel formation by highly biocompatible ionic gelation. We explored the ability of the platform to control the geometrical properties of hydrogel particles, including the length, aspect ratio, and curvature, which shape four geometries: L‐shaped continuous fibers, S‐shaped helical fiber, C‐shaped “tadpole” microfiber, and D‐shaped spherical particles.

## 2 Results and Discussion

### 2.1 Microfluidic Chip with Dynamic Liquid–Liquid Multiphase Interface for Multifaceted Fiber Production

**Figure 1:**
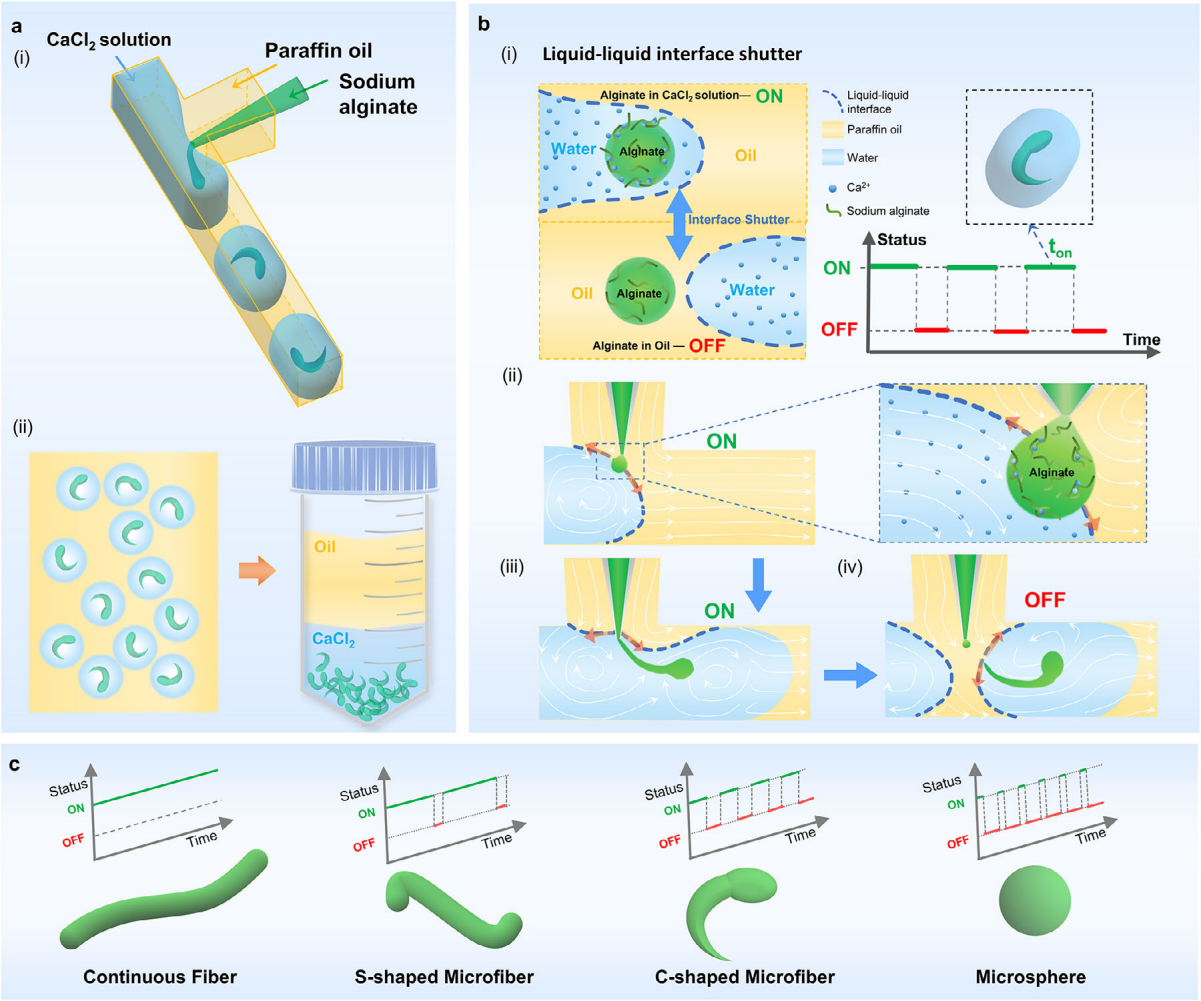
Programmable fabrication of hydrogel microfibers via a microfluidic liquid‐liquid interface shutter. a) Schematic of the microfluidic setup for generating alginate hydrogel microstructures. (i) A three‐phase flow of paraffin oil, CaCl_2_ solution, and sodium alginate is established in a hybrid T‐junction and co‐flow channel. (ii) Hydrogel microfibers form within monodisperse water‐in‐oil droplets and are recovered from the aqueous phase after phase separation. b) Schematic of the liquid‐liquid interface shutter mechanism. (i) The interface functions as a shutter, switching between ON (alginate‐Ca^2^⁺ contact and fiber elongation) and OFF (interface isolation). (ii‐iv) Sequential illustration of the interface dynamics during shutter‐mediated fiber formation, including coalescence of alginate solution with CaCl_2_ solution(ii), generation and elongation of microfibers (iii), abscission and encapsulation of alginate microfibers within CaCl_2_ droplets (iv). c) Morphological tuning of hydrogel products via programmed digital shutter control directs the formation of uninterrupted fibers, S‐shaped, tadpole‐like microfibers (C‐shaped), and spherical microspheres.

To precisely control the production of hydrogel fibers with diverse morphologies, we developed a microfluidic chip integrating a T‐junction and a co‐flow device, using three‐phase fluid streams of paraffin oil, calcium chloride solution, and sodium alginate solution (**Figure** **1**a; Figure S1c, Supporting Information). In the T‐junction, paraffin oil from the branch channel serves as the continuous phase (“oil”), while CaCl_2_ solution from the main channel forms the dispersed phase (“water”), generating controlled water‐in‐oil droplets—distinct from classical T‐junctions where the oil phase typically originates from the main channel.^[^ ^15^ ^]^ Simultaneously, the oil phase co‐flows with sodium alginate solution delivered through a capillary needle. Driven by capillary instability at the water‐oil interface, sodium alginate intermittently enters the CaCl_2_ droplets, forming an aqueous two‐phase system, where rapid ionic crosslinking with calcium ions yields stable alginate hydrogel microfibers. These microfibers are individually encapsulated within CaCl_2_ droplets and subsequently collected (Figure 1a ii).

The water‐oil interface can act as a dynamic digital shutter to make the sodium alginate alternatively contact and separate from the CaCl_2_ solution, which is defined as ON and OFF states separately. (Figure 1b‐i). In the ON state, the crosslinking of alginate by CaCl_2_ results in the formation of elongated fibers, whereas in the OFF state, the separation leads to dispersed drops. Detailed views of the interface dynamics further elucidate the shutter formation mechanism (Figure 1b(ii–iv)). The precise timing and control of these ON‐OFF states of the liquid‐liquid interface shutter are crucial for manipulating fiber morphology (Figure 1c). In the continuous ON state, elongated L‐shaped hydrogel fibers are formed. By adjusting the ON‐OFF duty cycle, we can generate S‐shaped microfibers, tadpole‐shaped fibers, and spherical droplets. Overall, the integration of the programmable digital shutter in our microfluidic chip facilitates the generation of different fiber morphologies.

### 2.2 Formation of Passive Digital Shutter by Controlling the Liquid–Liquid Interface

**Figure 2:**
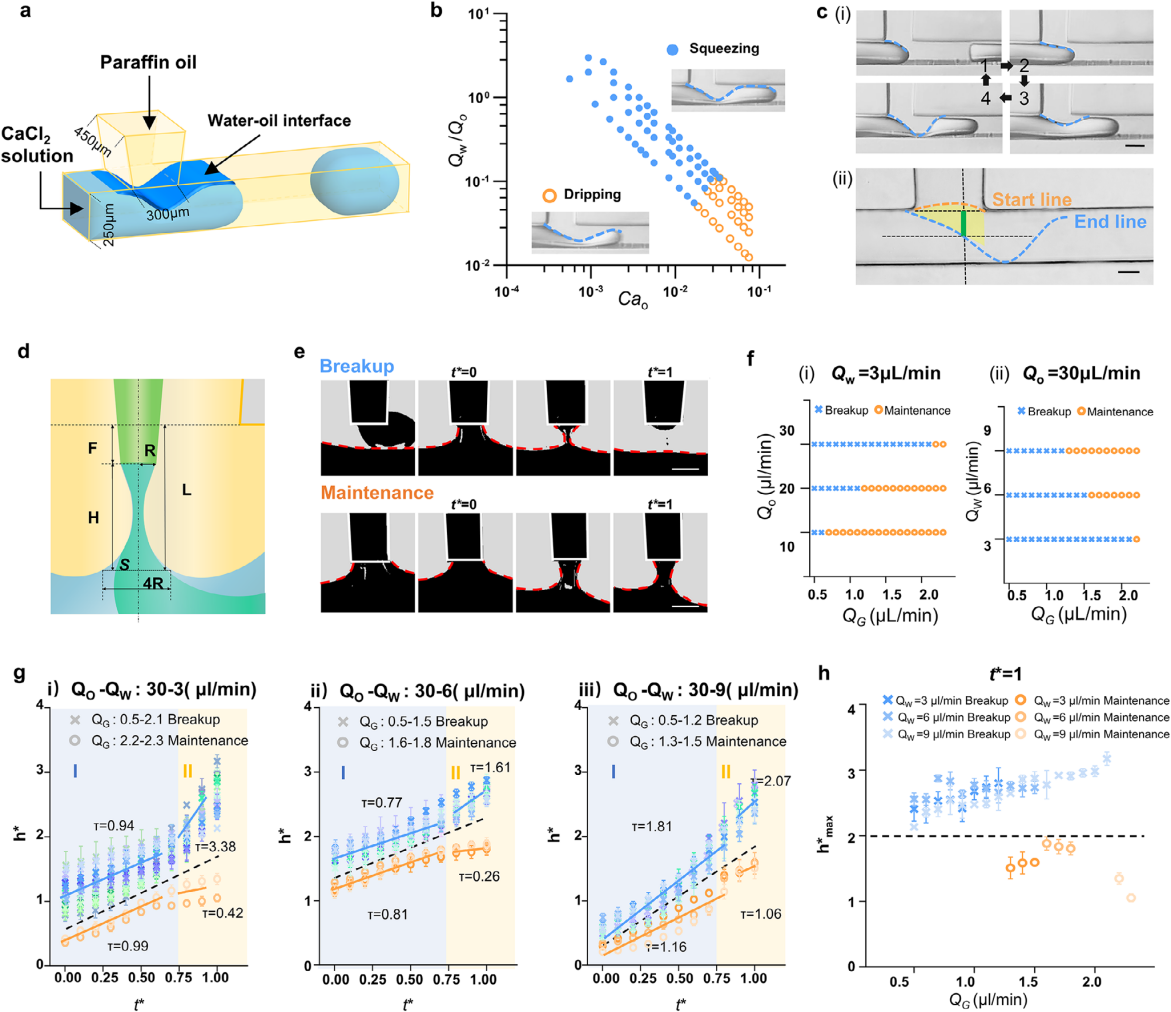
Design and formation of a passive digital shutter via liquid‐liquid interface manipulation. a) 3D schematic showing the formation of a water‐oil interface at the T‐junction, with paraffin oil injected through the main channel and CaCl_2_ solution through the branch channel. b) Flow regime map distinguishing the squeezing and dripping states as a function of the capillary number (Ca_o_) and the flow rate ratio (*Q*_w_ */Q*_o_), with representative micrographs showing the corresponding interface morphologies. c) Morphological characterization of the dynamic water‐oil interface during droplet generation. (i) Time‐resolved snapshots capture the shape transition from initial interface deformation to droplet detachment. (ii) Characteristic positions defining the feasible zone for capillary tip alignment (yellow area), with the determined insertion range highlighted in green. Scale bar: i) 200 µm; ii) 100 µm. d) Schematic illustrating the formation of a liquid bridge between the capillary tip and the surrounding water‐oil interface, where spatial parameters including tip radius (*R*), interposition length (*F*), separation distance (*H*), and the reference interface (*S*) are indicated. e) Time‐lapse images showing two distinct outcomes of the liquid bridge following the coalescence of alginate with CaCl_2_ solution: breakup (top) and maintenance (bottom). The red dashed line indicates the water–oil interface. Scale bar: 30 µm. f) Effect of flow rates on liquid bridge stability. Phase diagrams delineating breakup (blue) and maintenance (orange) regimes as a function of *Q*_g_ under fixed (i) *Q*_w_ = 3 µL/min and (ii) *Q*_o_=30 µL/min. g) Time evolution of the dimensionless length of the liquid bridge h* as a function of the characteristic time t* under different flow conditions: (i) *Q*_o_−*Q*_w_= 30−3 µL/min, (ii) *Q*_o_−*Q*_w_ = 30−6 µL/min, (iii) *Q*_o_−*Q*_w_ = 30−9 µL/min. Solid lines represent linear fits applied to distinct regimes, with the slope k indicating the scaling factor of bridge growth. The fitted k values are annotated in the plot. Data are presented as mean ± SD (*n* = 3). h) Statistical distribution of the limiting bridge length (h*_max_) at t* = 1. Data are presented as mean ± SD (*n* = 3).

Successful coalescence of sodium alginate from the branch capillary with the CaCl_2_ solution in the main channel depends on precise shaping and control of the water–oil interface at the T‐junction (**Figure** **2**a). To achieve this, the microfluidic chip was fabricated with a geometry tailored for predictable two‐phase flow: main and branch channels measuring 250 µm in width and 450 µm in height, and a junction width of 300 µm (Figure 2a; Figure S1, Supporting Information). In microfluidic two‐phase T‐junctions,^[^ ^23^, ^24^ ^]^ two distinct regimes are typically observed: squeezing and dripping. In the squeezing regime (Figure 2b; Movie S1, Supporting Information), the generated water droplet blocks the continuous oil‐phase channel and creates a periodically dynamic water–oil interface at the T‐junction,^[^ ^24^ ^]^ providing space for contact with the capillary needle. In addition, a pressure drop across the droplet^[^ ^25^ ^]^ may facilitate the pinch‐off of the alginate stream. In contrast, in the dripping regime (Figure 2b; Movie S2, Supporting Information), the water phase only partially occupies the channel, and the water‐oil interface is positioned downstream of the T‐junction. Therefore, the interface in the squeezing regime is more suitable as a passive digital shutter and is adopted in this study. A phase diagram of the squeezing and dripping regimes is shown in Figure S4a (Supporting Information) as a function of water and oil flow rates, and further transformed in Figure 2b into capillary numbers *Ca_o_* = µ_*o*_ *U_o_*/γ and flow rate ratios, φ = *Q*_w_/*Q*_o_, where µ_*o*_, *U*_o_ and *Q*_o_ are the viscosity, the flow speed and the volumetric flow rate of oil (liquid paraffin), γ is the interfacial tension, and *Q*_w_ is the volumetric flow rate of water (CaCl_2_ solution). As the capillary number increases beyond a critical value (∼0.01‐0.03), a transition from squeezing to dripping occurs, consistent with previous numerical estimates (∼0.015).^[^ ^23^ ^]^ Notably, the channel aspect ratio (*AR = height/width*) strongly influences the squeezing–dripping behavior. Comparison of chips with heights of 250 µm (*AR* = 1.0) and 450 µm (AR = 1.8) shows that higher AR reduces the local flow velocity and effective *Ca*_o_, shifting the squeezing–dripping transition toward higher oil flow rates and widening the squeezing regime (Figure S4b,c; Figure S10, Supporting Information), consistent with previous scaling laws.^[^ ^26^, ^27^ ^]^ Consequently, the 450 µm‐height chip provides a broader operational window.

The profile of the dynamic water‐oil interface (Figure 2c‐i) was further characterized to determine the space available for the capillary tip. The interface cross‐section resembles a slightly convex parabola at the onset of squeezing and a groove‐like concave parabola at the end point (Figure 2c‐ii). Both parabolas are defined by three characteristic points each and were quantitatively measured under varying *Q*_w_ and *φ* (Figure S5i,ii, Supporting Information). The range of the determined interposition of the capillary tip is marked in green and located at the intersect region of the two parabolas in yellow (Figure 2c‐ii; Figure S5iii,iv, Supporting Information).

The extrusion of alginate forms a co‐flow with the continuous phase. As the flow advances, alginate contacts the CaCl_2_ solution, generating a liquid bridge. As shown in Figure 2d, the alginate bridge is captured between the capillary tip (radius *R*, interposition length *F*) and the water‐oil interface, separated by a distance *H* from each other. Upon coalescence with the CaCl_2_ solution, the alginate liquid bridge undergoes two possible scenarios: either breakup via ductility, capillary thinning, and pinch‐off (Figure 2e; Movie S3, Supporting Information), or stable maintenance without rupture (Figure 2e; Movie S4, Supporting Information). In the ductility stage, the liquid bridge extends axially and contracts radially, with the speed of the water‐oil interface within a certain range. Driven by the interfacial tension between the alginate solution and oil, the system then enters the capillary thinning stage, where further radial contraction occurs. Finally, when the length of the liquid bridge is long enough, it enters the pinch‐off stage, and the liquid bridge breaks. The red dashed lines in Figure 2e represent the dynamically captured interfaces under both breakup and stable maintenance scenarios. Two or more tiny satellite drops are observed subsequent to liquid bridge breakup, which reflects the nonlinear features of breakup.^[^ ^20^, ^28^ ^]^ We further reveal the influence of *Q*_o_, *Q*_w,_ and *Q*_g_ (the volumetric flow rate of alginate) on the maintenance and breakup dynamics of the liquid bridge (Figure 2f). Increasing *Q*_o_ promotes breakup (Figure 2f‐i), while higher *Q*_w_ and *Q*_g_ have a stabilizing effect on the liquid bridge (Figure 2f‐ii). To quantify the characteristics of liquid bridge maintenance and breakup, we measured the dimensionless bridge length h* = *h*/*R* and plotted its evolution against normalized time t* = *t*/*T* under varying flow rates (Figure 2g; Figure S6, Supporting Information). Both stable and breaking bridges show increasing h* with t*, but follow distinct two‐regime scaling patterns. In unbroken cases, regime I shows a higher scaling factor *k*, slowing h* growth in regime II; whereas for breaking bridges, regime II exhibits a higher scaling factor, accelerating h* growth. Notably, when the limiting length or the maximum length attained by the bridge (h*_max_) exceeds ≈2‐which is consistent for both 450 µm and 250 µm high, the liquid bridge consistently breaks, providing a clear criterion for controlling the shutter formation (Figure 2h; Figure S7, Supporting Information).

### 2.3 Programmable Digital Shutter for Fabrication of Multifaceted Microfiber Structure

**Figure 3:**
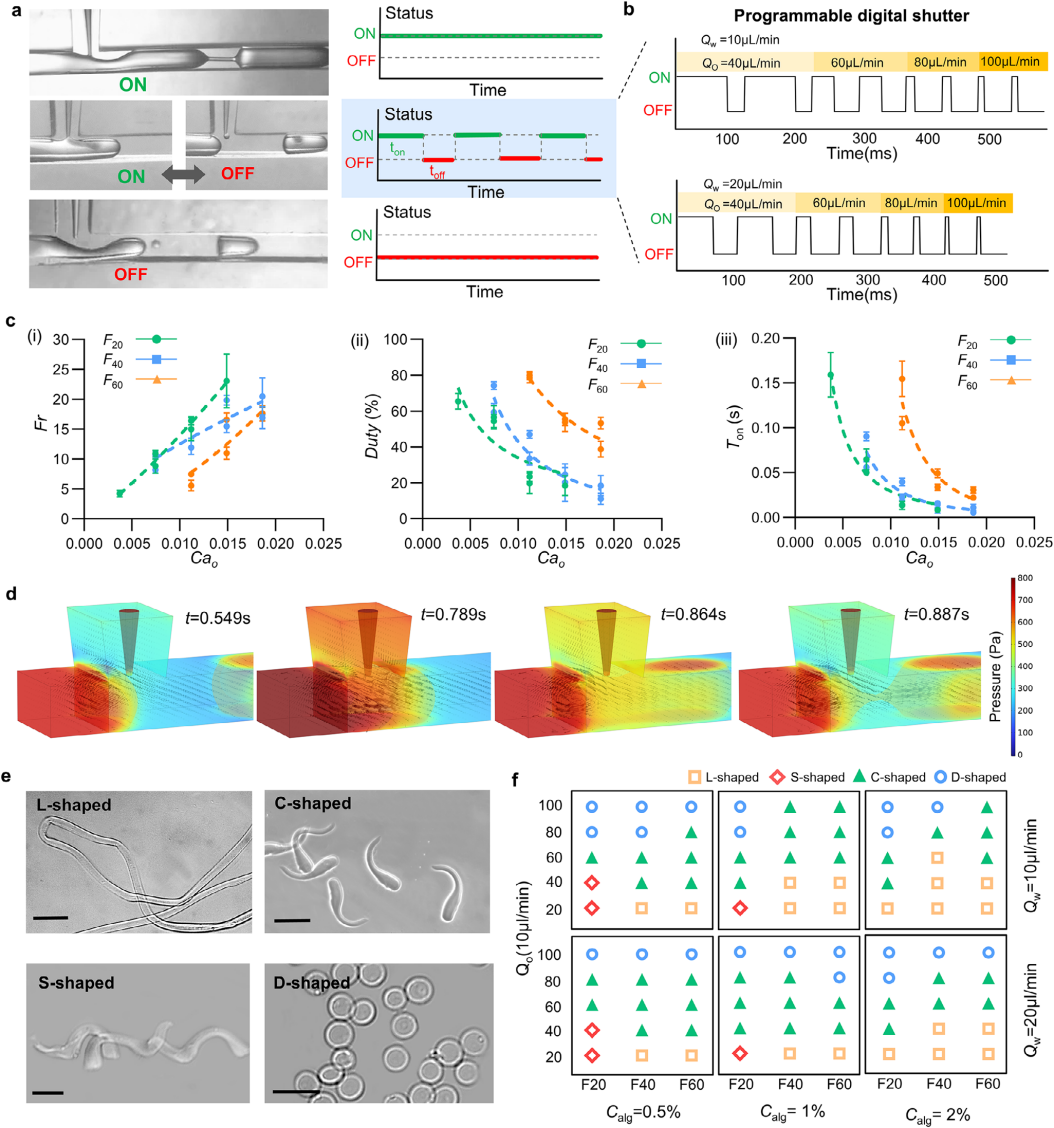
Programmable modulation of hydrogel microfiber morphology via flow‐regulated passive digital shutter. a) Schematic illustration of the microfluidic shutter operating in three distinct modes: continuous ON, periodic ON‐OFF, and continuous OFF. b) Representative time sequences of shutter activation under different oil‐phase flow rates. Precise control over the switching cycle is achieved by tuning the flow rate, enabling programmable actuation. c) Quantitative characterization of the influence of capillary number (Ca_o_) and interposition length (*F*) on shutter regulation. (i) The production frequency (*Fr*) as a function of Ca_o_ for different interposition lengths (*F*_20_, *F*_40_, and *F*_60_). (ii) *Duty* as a function of Ca_o_ for different interposition length (*F*_20_, *F*_40_, and *F*_60_). (iii) The shutter activation time (*T*_on_) as a function of Ca_o_ for different interposition lengths (*F*_20_, *F*_40_, and *F*_60_). Data are presented as mean ± SD (*n* = 20–30). d) 3D numerical simulation of multiphase flow showing the effect of needle insertion on digital shutter formation. Black volume arrows denote the flow field, and color indicates pressure distribution. e) Representative images showing four distinct microfiber morphologies: continuous fibers (L‐shaped), helical fiber (S‐shaped), "tadpole" microfiber(C‐shaped), and spherical particles (D‐shaped). Scale bar: 100 µm. f, Phase diagram mapping the relationship between microfiber morphology and experimental parameters, including flow rates (*Q*_w_, *Q*_o_), interposition length (*F*), and sodium alginate concentration.

Programming the passive digital shutter enables precise control over the alginate gelation process, allowing the fabrication of hydrogel microfibers with tunable lengths and morphologies. The system operates in three distinct modes‐continuous ON, periodic ON‐OFF, and continuous OFF, offering specific control over microfiber formation (**Figure** **3**a). In continuous ON mode, uninterrupted gelation produces elongated fibers (Movie S5, Supporting Information). In continuous OFF mode, gelation ceases, and alginate droplets disperse in oil and flow downstream (Movie S6, Supporting Information). The periodic ON‐OFF mode regulates gelation cycles, yielding monodisperse hydrogel structures (Movie S7, Supporting Information). Thus, microfiber length and production frequency (*Fr*) can be precisely tuned by adjusting the ON‐state duration and duty cycle. As demonstrated in the previous section, Ca_o_ significantly affects liquid‐liquid interface dynamics, while the capillary tip interposition length (F) directly relates to h*. Thus, the production frequency *Fr* = 1/*T*_on_ and duty cycle (*duty* = *T*_on_/*T*_all_) of microfibers can be predictably controlled by adjusting Ca_o_ and *F*. The production frequency (*Fr*) shows an approximately linear dependence on different *F* (*F*_20_, *F*_40_, *F*_60_) (Figure 3c‐i), consistent with previous reports on droplet production frequency in T‐junctions under the squeezing regime.^[^ ^29^ ^]^ In contrast, the duty cycle decays exponentially across all F (Figure 3c‐ii). The dotted lines represent power‐law fits: $\alpha Ca_{o}^{\beta }$, where α is a fitting parameter and β is the power‐law exponent, ranging from −0.78 to −1.51 as *F* varies (Figure 2d). This dual‐parameter coordination enables precise control of the shutter activation time (*T*_on_), which also decays exponentially with Ca_o_ increasing at all F, with β ranging from −1.75 to −3.59 (Figure 3c‐iii). As the capillary tip is inserted deeper into the main channel, the decay rate becomes more pronounced due to the increasingly confined space for the interface to retreat to h*_max_. When Ca_o_ exceeds 0.15, the effect of capillary tip interposition becomes negligible, approaching the transition from the squeezing to dripping regime. To further investigate the effect of needle insertion on digital shutter formation, multiphase flow was simulated using the Level Set method. As shown in Figure 3d and Figures S8–S11 and Movie S8 (Supporting Information), during the capillary thinning and pinch‐off phases of the liquid bridge, elevated pressure and shear rate at the capillary tip outlet accelerate its rupture. Additionally, periodic variations in pressure and shear rate induce a repeated thinning‐breakup cycle, resulting in periodic detachment of the liquid bridge (Figure S11b, Supporting Information). The pressure gradient dynamics are similar to those observed in T‐junctions without needle insertion,^[^ ^25^ ^]^ indicating that needle insertion has a limited influence on water‐oil interface behavior.

To explore the influence of the passive digital shutter on microfiber morphology, we programmed flow rate ratios by fixing *Q*_w_ at 10 and 20 µL/min, while modulating *Q*_o_ (20‐100 µL/min), adjusting the capillary tip interposition length (20, 40, 60 µm), and varying alginate concentrations (0.5%‐2%). Four distinct morphologies were obtained (Figure 3e): continuous fibers (L‐shaped), helical fibers with curvature and torsion (S‐shaped), tadpole‐like microfibers with curvature and aspect ratio (C‐shaped), and spherical, droplet‐like particles (D‐shaped). The phase diagram in Figure 3f illustrates microfiber morphology as a function of *Q*_o_, *Q*_w_, *F*, and alginate concentration. L‐shaped fibers form predominantly under continuous ON conditions, where uninterrupted gelation generates stable fibers. At 2% alginate concentration, the distribution of L‐shaped fibers is widest, as increased viscosity stabilizes the liquid bridge. S‐shaped fibers are rare and occur only under low *Q*_o_ and shallow needle insertion. C‐shaped microfibers, exhibiting tadpole‐like curvature, are the most prevalent morphology. Their distribution window narrows as alginate concentration increases, highlighting the role of viscosity in affecting the maximum length attained by the liquid bridge (h*_max_) and hence controlling fiber geometry. D‐shaped microspheres form under continuous OFF conditions, where gelation occurs downstream. Additionally, when the activation time is extremely brief, leading to immediate rupture upon alginate contact with CaCl_2_ droplets, spherical particles also emerge.

Notably, even when *Q*_o_, *Q*_w_, *F*, and alginate concentration are fixed, varying the alginate flow rate (*Q*_G_) alone can induce a transition in microfiber morphology from D‐ to C‐ to L‐shaped fibers (Figure S12, Supporting Information). Increasing *Q*_G_ clearly extends the length of the microfiber, and once stable fibers are formed, further increases in *Q*_G_ also lead to larger fiber diameters (Figure S13, Supporting Information). This indicates that modulation of *Q*_G_ provides an additional programmable means to control both the length and diameter of microfibers. In summary, our results demonstrate that programmable control of the digital shutter‐through modulation of *Q*_o_ and capillary tip positioning‐precisely governs gelation dynamics, enabling the fabrication of diverse microfiber structures.

### 2.4 Geometrical Modulation of C‐Shaped Microfibers

**Figure 4:**
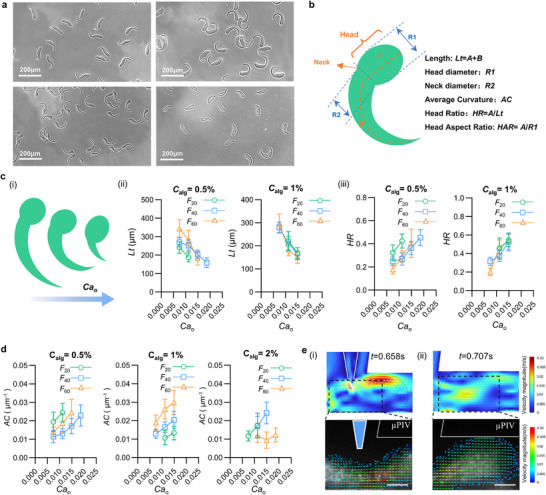
Geometric modulation of C‐shaped microfibers. a) Optical micrographs of representative C‐shaped hydrogel microfibers exhibiting tadpole‐like morphologies with varying lengths and curvatures. b) Schematic illustration defining the geometric descriptors used for quantitative analysis of C‐shaped microfiber morphology. c, (i) Conceptual illustration of progressive shortening and head prominence with increasing Cao. Total fiber length (Lt) (c ii), head ratio (HR) (c iii), and average curvature (AC) (d)as a function of Cao, alginate concentrations (w/v), and interposition length (F_20_, F_40_, and F_60_). Data are presented as mean ± SD (*n* = 50–100). e, Flow field analysis during microfiber formation from numerical simulations and experimental µPIV measurements. (i) Velocity magnitude is indicated by color, and flow direction is shown by arrows in the alginate injection region, revealing the formation of strong localized vortices. The arrow length is proportional to the local flow velocity. (ii) Internal flow inside the CaCl_2_ droplet encapsulating the C‐shaped microfiber, showing vortex structures. Color represents velocity magnitude, and arrows indicate flow direction and speed. Scale bar: 100 µm.

Compared to the other three microfiber types, C‐shaped hydrogel microfibers are rarely reported and represent particularly anisotropic microparticles. They exhibit a distinctive tadpole‐like geometry, resembling vertebrate embryos in early development, with a clearly defined head and slender tail, and show variations in length and curvature (**Figure** **4**a). Therefore, their morphological characteristics, controlled by the programmed passive digital shutter, were systematically investigated. To quantitatively analyze these variations, several geometric parameters were evaluated, including total length (*Lt*), head diameter (*R1*), neck diameter (*R2*), average curvature (*AC*), head ratio (*HR*), and head aspect ratio (*HAR*) (Figure 4b). These parameters change with increasing Ca_o_, leading to distinct fiber dimensions and shapes (Figure 4c‐i). The *Lt* ranges from ∼150 µm to 350 µm and decreases as Ca_o_ rises, with a sharper decline observed as the interposition length increases (Figure 4c‐ii). This trend aligns with the exponential decay of *T_on_* in response to both Ca_o_ and *F*. Notably, the length reduction is mainly due to tail shortening, while the head remains relatively stable, causing *HR* to increase at higher Ca_o_ (Figure 4c‐iii). This highlights that liquid bridge maintenance time primarily governs length variation. Importantly, this effect is less pronounced at higher alginate concentrations, likely due to viscosity‐induced stabilization (Figure S14, Supporting Information).

In contrast to total fiber length, the head (*R1*) and neck (*R2*) diameters exhibit position‐dependent variations rather than a uniform dependence on Ca_o_ or alginate concentration. A significant positional effect is observed: R1 is consistently the largest at *F*_20_ compared to *F*_40_ and *F*_60_ (Figure S15i, Supporting Information), suggesting that this position favors broader droplet coalescence and larger meniscus formation. Conversely, *R2* reaches a minimum at *F*_40_ (Figure S15ii, Supporting Information). Moreover, head morphology is more sensitive to alginate concentration, with microfibers formed at 2% alginate showing a higher *HAR* than those at 0.5% or 1% (Figure S16, Supporting Information).

The average curvature (*AC*) of C‐shaped microfibers increases with rising Ca_o_, ranging from ≈0.009 to 0.025 µm^−1^, indicating enhanced bending at higher Ca_o_, especially at lower alginate concentrations (Figure 4d). As curvature is the unique feature of C‐shaped microfibers, we explored its formation mechanism via numerical simulations and micro‐particle image velocimetry (µPIV) (Figure 4e). The velocity vector fields reveal a distinct vortex at the alginate injection region during fiber formation, which likely induces bending (Figure 4e, i). Additionally, a vortex persists inside the droplet containing the fiber post‐formation (Figure 4e‐ii), and its chaotic advection as the droplet moves through the microchannel may further enhance curvature.^[^ ^30^ ^]^ To validate these observations, µPIV measurements were compared with numerical simulations, showing agreement within 20% (Figure S17, Supporting Information),^[^ ^31^ ^]^ confirming the reliability of the measured velocity fields. During neck contraction of the CaCl_2_ droplet, internal velocity at the neck gradually rises to a maximum, promoting CaCl_2_ transfer into the droplet and increasing the velocity gradient, which favors vortex formation.

### 2.5 Fabrication of Cell‐Laden and Magnetic‐Responsive C‐Shaped Microfibers

**Figure 5:**
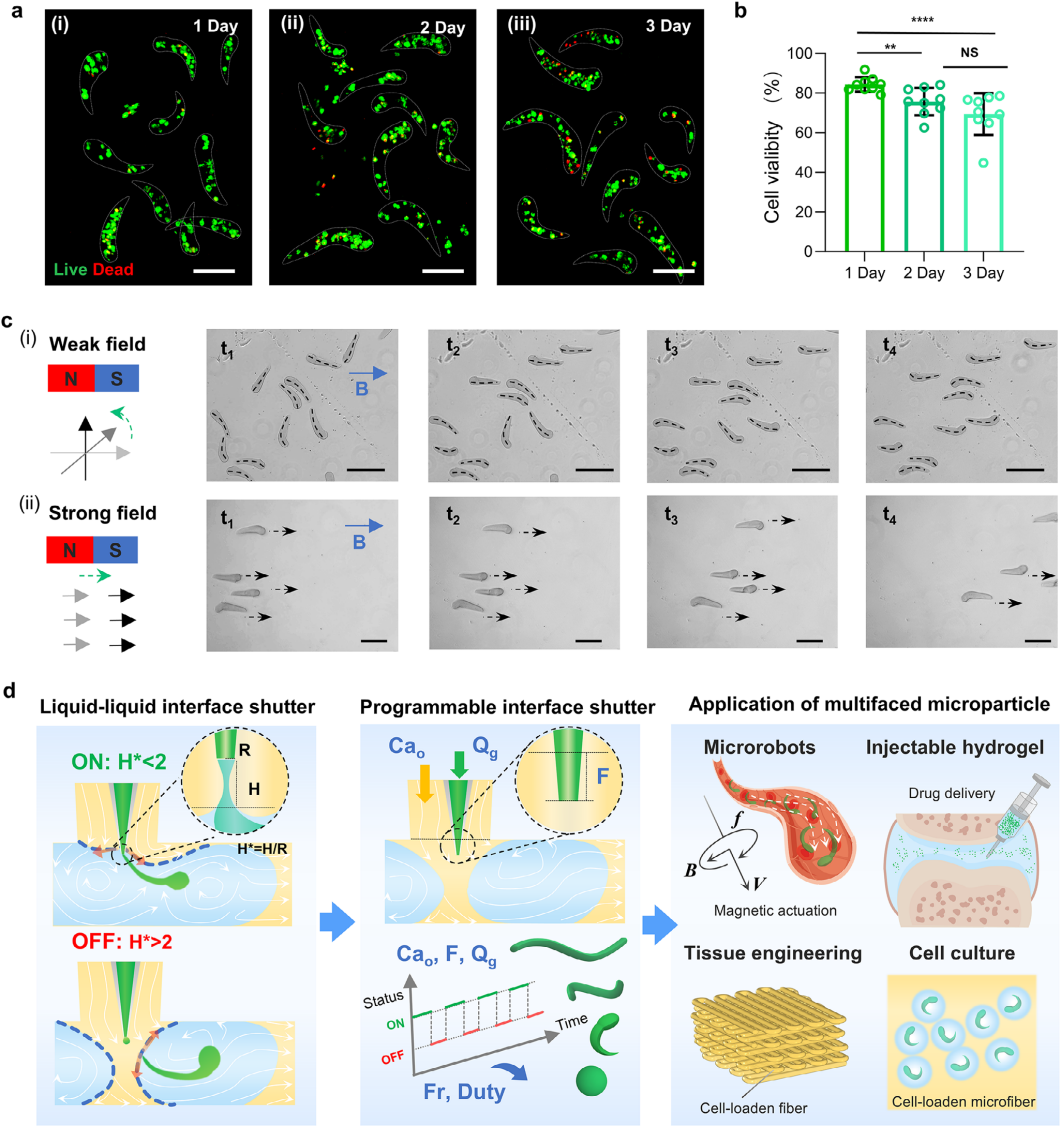
Fabrication and evaluation of cell‐laden and magnetic‐responsive C‐shaped microfibers. a). Confocal microscopy images showing cell viability within the microfibers at 1, 2, and 3 days after fabrication (cell density: 3 × 10⁶ cells mL^−1^). Scale bar: 100 µm. b) Quantitative analysis of cell viability over 3 days post‐encapsulation. Data are presented as mean ± SD (*n* = 9). Statistical significance was determined by one‐way ANOVA followed by Tukey's post‐hoc test (^**^ *p* < 0.01, ^****^ *p* < 0.0001). c) Magnetic responsiveness of Fe3O4 nanoparticles ‐embedded C‐shaped microfibers. Representative images show (i) reorientation at 4 cm and (ii) directional motion at 1 cm under magnetic fields. Scale bar: 200 µm. d) Overview of the programmable interface shutter system, showing formation of the liquid–liquid interface shutter, programmable shutter operation for microfiber fabrication, and potential applications of the fabricated microfibers.

To demonstrate the potential of this microfluidic system, we fabricated cell‐laden C‐shaped microfibers and magnetically responsive ones. As a proof of concept, ultrafine C‐shaped hydrogel microfibers encapsulating endothelial cells were first biofabricated, which can act as modular building blocks for vascularized tissues. As illustrated in Figure S18a (Supporting Information), endothelial cells suspended in alginate solution were extruded through the microfluidic chip, where the digital shutter at the liquid‐liquid interface enabled fiber formation, followed by washing and culture. To assess the effect of cell density on fiber morphology, cells were encapsulated at 3 × 10^6^, 5 × 10^6^, and 7 × 10^6^ cells mL^−1^. At 3 × 10⁶ cells mL^−1^, fibers maintained stable C‐shaped morphology, with viability slightly decreasing from 84.3% ± 3.7% on day 1 to 69.4% ± 10.1% on day 3 (**Figure** **5**a,b), indicating generally good survival over the short term. At 7×10^6^ cells/mL, severe structural disruption and capillary clogging occurred, indicating a critical density threshold for encapsulation. Increasing cell density to 5 × 10⁶ and 7 × 10⁶ cells mL^−1^ resulted in irregular fiber shapes, structural disruption, and lower viabilities (69.6% ± 8.4% and 52.2% ± 8.4%, respectively), suggesting a critical threshold beyond which mechanical confinement and intercellular compression during extrusion compromise both fiber integrity and cell survival (Figure S18b,c, Supporting Information). This reduction was attributed to elevated shear stress during extrusion, caused by mechanical confinement and intercellular compression at high cell density. These observations can be attributed to extrusion‐induced mechanical stresses. At a constant f *Q*_G_, shear and extensional stresses increase with cell density, raising the pressure work and shear stress (τ = 4ηU/R), which in turn elevates the damaged cell ratio.^[^ ^32^ ^]^ At low cell densities, stresses remain moderate, preserving fiber morphology and viability, whereas at higher densities, mechanical confinement amplifies stresses, causing structural disruption and reduced survival. Decreasing alginate concentration or lowering *Q*_G_ could reduce shear‐induced damage and enhance cell viability. As another proof of concept, Fe_3_O_4_ nanoparticles were embedded to fabricate magnetic‐responsive C‐shaped microfibers. Upon exposure to a weak magnetic field (magnet at 4 cm), the microfibers reoriented toward the magnet. Increasing the field strength (magnet at 1 cm) induced directional motion toward the magnetic source (Figure 5c).

## 3 Conclusion and Future Perspective

Here we present a programmable passive digital shutter mechanism based on multiphase liquid–liquid interface dynamics, enabling precise fabrication of multifaceted hydrogel microfibers on a microfluidic platform. In contrast to traditional microfluidic platforms that implement logic gates or transistor‐like behaviors through structural design or pressure control,^[^ ^33^, ^34^, ^35^, ^36^ ^]^ we leverage capillary instabilities between immiscible fluids to realize fluidic logic without external actuation. By tuning interface dynamics and fluid parameters, logic functions emerge as discrete variations in microfiber geometry and production frequency, directly encoding shutter logic. This approach enables programmable control of fluid interfaces, offering a template‐free, reconfigurable, and low‐energy strategy for microfluidic regulation, thereby broadening the design landscape for fluidic logic systems. Beyond its conceptual advance, the system's versatility and biocompatibility highlight its potential for diverse applications. Magnetically responsive microfiber hydrogels fabricated via this method exhibit shape‐morphing under external fields, making them ideal for soft robotic systems^[^ ^37^ ^]^ and minimally invasive therapeutics.^[^ ^38^ ^]^ Furthermore, the tunable morphology and spatial patterning enabled by shutter logic provide new avenues for engineering hierarchical scaffolds tailored to vascularization, neural guidance, and localized drug delivery.^[^ ^39^, ^40^, ^41^ ^]^ Figure 5d schematically summarizes this concept, illustrating the formation and control of the interface shutter, the programmable fabrication of microfibers with distinct geometries, and their potential applications across biomedical and soft robotic fields.

Another important aspect of the system lies in the mechanistic origin of the diverse hydrogel microfiber morphologies. These morphologies arise from the interplay between liquid‐bridge stability and intradroplet hydrodynamics. When the liquid bridge remains stable, capillary forces dominate over Rayleigh–Plateau instabilities, enabling continuous elongation into linear fibers. As the bridge approaches the critical breakup point, vortical recirculation develops within the CaCl_2_ droplets, generating non‐uniform axial stresses that bend or twist the soft hydrogel ligament, giving rise to helical or curved (S‐ and C‐shaped) fibers.^[^ ^42^, ^43^ ^]^ In contrast, rapid rupture events inhibit axial extension, yielding D‐shaped microspheres instead.^[^ ^44^ ^]^ Such morphological transitions reflect the coupled effects of capillary‐driven thinning, viscous stresses, and droplet‐scale vortex dynamics, consistent with the nonlinear pinch‐off physics of viscous jets and regime transitions in confined microfluidic dripping‐to‐jetting flows.^[^ ^45^, ^46^ ^]^ Together, these phenomena provide a mechanistic rationale for the observed fiber geometries and support the programmable control of microfiber structures via digital shutter logic.

While the liquid‐liquid interface dynamic shutter mechanism has shown remarkable performance in generating hydrogel microfibers with various anisotropic shapes, some limitations persist. One is the lack of making the anisotropic microfibers with different components along the fiber, which may be realized by injection of multi‐materials from co‐flow devices instead of only the alginate solution^[^ ^47^ ^]^ and may have plenty of potential applications, such as biohybrid robots^[^ ^48^ ^]^ and microscale droplet‐based iontronics.^[^ ^49^ ^]^ Additionally, the effects of rheological properties on the breakup of the bridge alginate remain insufficiently explored,^[^ ^50^ ^]^ especially under complex hydrogel systems and cell encapsulation conditions. Moreover, achieving high‐throughput production and repeated use of the microfluidic chip, including maintaining surface hydrophobicity for consistent operation, remains a practical challenge for scalable manufacturing. Finally, integrating this liquid‐liquid interface‐driven, energy‐free logic with emerging micromanufacturing techniques, such as 3D bioprinting^[^ ^51^, ^52^ ^]^ or responsive material platforms,^[^ ^53^ ^]^ could further extend its utility in tissue engineering and colloidal robotics.

## 4 Experimental Section

### Materials

Calcium chloride (CAS‐No: 10035‐04‐8), sodium alginate (CAS‐No: 9005‐38‐3), and the photoinitiator Irgacure 2959 (CAS‐No: 106797‐5309) were purchased from Sigma‐Aldrich. Liquid paraffin oil (CAS‐No: 8042‐47‐5), Fe_3_O_4_ magnetite nanoparticles (CAS‐No: 1317‐61‐9), and Span 80 (CAS‐No: 1338‐43‐8) were supplied by Aladdin. GelMA was synthesized in our laboratory according to the protocol described in a previous study.^[^ ^54^ ^]^ Deionized water (resistivity: 18.2 MΩ·cm^−1^) was prepared using a Millipore Milli‐Q system. Endothelial cell medium was obtained from ScienceCell, and the Live/Dead Viability/Cytotoxicity Kit was acquired from Thermo Fisher Scientific.

### Microfluidic Device Fabrication

The microfluidic device was constructed by integrating a T‐junction microchannel with a tapered glass capillary (Figure S1 c, Supporting Information). The T‐junction microchannel was fabricated using standard photolithography to produce a silicon wafer mold (Figure S1 a, Supporting Information). A polydimethylsiloxane (PDMS) precursor (Sylgard 184, Dow Corning) was prepared by mixing the base and curing agent at a 10:1 weight ratio, poured onto the mold, and cured at 80 °C for 2 h (Figure S1 b, Supporting Information). After curing, the PDMS replica was peeled off from the mold, and inlet and outlet ports were created using a 2 mm biopsy punch. The PDMS channel layer and a glass slide were treated with oxygen plasma (ETP) for 60 s to activate the surfaces, aligned, and irreversibly bonded. The assembled device was baked at 80 °C for 30 min to enhance bonding strength. Tapered glass capillaries were fabricated using a micropipette puller (Sutter P‐97). Commercial glass capillaries (outer diameter: 1 mm; inner diameter: 0.8 mm) were pulled to produce tips with an inner diameter of ≈30 µm and an outer diameter of ≈40 µm.

For device assembly, the tapered glass capillary was inserted into the side port of the T‐junction channel and positioned at the desired location. The positioning process was conducted under a microscope using an XYZ positioning stage equipped with a capillary holder (Figure S2, Supporting Information). The T‐junction region was imaged under the microscope and aligned with a predefined reference point. The capillary tip was finely adjusted using the XYZ positioning stage according to the desired insertion depth and alignment reference, with a positioning tolerance of ±3 µm in our experiments. After carefully inserting the glass capillary into the side port, it was secured in place using the holder. Subsequently, a small amount of epoxy resin was applied around the side opening to seal the junction, ensuring structural stability and preventing leakage. The device was left undisturbed until the epoxy resin was fully cured before use.

### Microfluidic Platform Setup and Microfiber Fabrication

A microfluidic platform was established, consisting of a microfluidic chip, syringe pumps, and a microscope (Figure S3, Supporting Information). The system was designed to enable precise flow rate control of three immiscible phases: calcium chloride solution, paraffin oil, and sodium alginate solution. Each phase was independently injected into the microfluidic chip via polyethylene (PE) tubing (outer diameter: 2.0 mm; inner diameter: 0.8 mm) using syringe pumps. Within the microfluidic chip, CaCl_2_ droplets encapsulated the forming microfiber structures, which were subsequently collected at the outlet channel. After collection, the paraffin oil and CaCl_2_ solution were allowed to separate under gravity, and the upper paraffin oil layer was carefully removed. Then the collected microgels were repeatedly washed with deionized water to eliminate residual oil. This purification step ensured the production of well‐defined microfibers with controlled dimensions and composition, suitable for further experimental applications. For the fabrication of endothelial cell‐laden microfibers, all solutions were sterilized using 0.22 µm filters (Millipore, USA) prior to use, and all microfluidic chips and tools were disinfected. Human umbilical vein endothelial cells (HUVECs) were suspended in a mixture of 0.5% (w/v) sodium alginate and 7.5% (w/v) GelMA at final cell densities of 3 × 10⁶, 5 ×1 0⁶, or 7 × 10⁶ cells mL^−1^. The resulting cell‐laden microfibers were collected at the outlet and incubated in phosphate‐buffered saline (PBS) at 37 °C. During the encapsulation process, a constant supply of warm CaCl_2_ solution was added to stabilize the structure, and the supernatant was subsequently removed. The entire process was completed within 2 h at room temperature (22–25 °C), and the microfibers were transferred into fresh culture medium every 20–30 min to maintain cell viability. After washing the cell‐laden fibers thoroughly with endothelial growth medium to remove residual calcium ions and paraffin oil, the microfibers were exposed to UV light (6.9 mW cm^−2^) for 35 s to induce GelMA crosslinking. The final cell‐encapsulating microfibers were cultured in suitable endothelial cell culture medium for downstream applications.

For the preparation of Fe_3_O_4_ nanoparticle‐loaded microfibers, a 0.5% (w/v) sodium alginate solution was mixed with Fe_3_O_4_ nanoparticle suspension at a volume ratio of 1 mL sodium alginate solution to 10 µL nanoparticle dispersion. The mixture was injected into 0.2 mol L^−1^ CaCl_2_ solution to trigger gelation, forming continuous microfibers. After collection, residual paraffin oil was removed through washing, and the purified fibers were subjected to subsequent magnetic manipulation experiments.

### Imaging and Analysis of Oil‐Water Interface Dynamics

The dynamics of the water‐oil interface within the T‐junction microfluidic chip were captured using the slow‐motion feature of a Huawei Mate30 smartphone, which recorded video at a frame rate of 960 fps for a continuous duration of 0.5 s. This temporal resolution was sufficient to document the complete cycle of interface deformation and rupture under varying flow rates. Post‐acquisition, each frame was extracted from the video using a custom MATLAB script. These frames were then analyzed with Fiji (an open‐source distribution of ImageJ, NIH) to track the position of the oil‐water interface. Specifically, images corresponding to the initial and final states of the periodic oil‐water interface were selected. Within these images, the coordinates of key feature points‐designated as A, B, and P‐were determined based on a pre‐established coordinate system.

### Image Analysis of Liquid Bridge Dynamics

High‐speed videos of the liquid bridge stretching process were recorded using an OLYMPUS i‐SPEED 3 camera at 15000 frames per second (fps) and analyzed in Fiji. Original AVI files were imported and converted from RGB to 8‐bit grayscale to reduce computational complexity. Image contrast and brightness were adjusted, followed by binarization and edge detection to enhance the liquid bridge contour. A reference line was drawn along the central axis of the capillary needle, and the position of the liquid surface was manually identified in each frame. The vertical distance between the liquid surface and the capillary tip was measured as the instantaneous bridge height H. The time axis was defined such that *t* = 0 corresponds to the initial symmetric equilibrium state, and absolute time t was computed from frame indices and the known frame rate. The rupture point of the bridge was marked as T, indicating the total stretching duration. To allow standardized comparisons across different experimental conditions and eliminate absolute scale effects, the bridge height and time were nondimensionalized as h*  =  *h*/*R* and t* = *t*/*T*, where R is the radius of the capillary tip.

### Morphology Analysis of C‐Shaped Microfiber

The morphology of C‐shaped microfibers was quantitatively analyzed using bright‐field images obtained under varying conditions with a Leica phase contrast microscope. Images were captured for microfibers of different sizes and preparation conditions. Morphological measurements, including microfiber length, head diameter, neck diameter, head length, and average curvature, were conducted using ImageJ software. The average curvature was specifically analyzed using the curvature analysis plugin, Kappa, available in Fiji.

### Numerical Simulation of Two‐Phase Flow

A numerical model for two‐phase flow was developed based on the level‐set method to simulate the dynamic evolution of the liquid–liquid interface in a microfluidic T‐junction geometry. The model resolved the interaction between the aqueous and oil phases co‐flowing in the T‐junction channel, capturing the interface deformation and the associated flow field. All simulations were performed using COMSOL Multiphysics 6.0.

To simplify the model and ensure consistency with the two‐phase flow framework, the injection of sodium alginate solution in the experiments was approximated by CaCl_2_ solution in the simulations. Two microchannel configurations with heights of 250 and 450 µm were constructed to match the experimental chip geometries. The geometric dimensions and boundary conditions of the computational domain are provided in Figure S8 (Supporting Information). The fluid properties used in the simulations were determined from experimental measurements: the viscosities of the CaCl_2_ solution and paraffin oil were set to 3.25 mPa·s and 65 mPa·s, and the corresponding densities were 1.106 and 0.86 g cm^−3^, respectively. The interfacial tension coefficient between the two immiscible phases was 0.0505 N m^−1^.^[^ ^55^ ^]^ Consistent with the experimental setup, the CaCl_2_ solution was injected through the capillary at 1 µL min^−1^ and through the aqueous channel at 10 µL min^−1^, while the oil phase flow rate was varied at 20, 40, and 60 µL min^−1^. The model successfully captured the dynamic interface evolution, pressure field, and velocity distribution under these flow conditions, allowing direct comparison with experimental observations.

To ensure numerical accuracy, mesh and time‐step independence analyses were performed prior to the main simulations. The yellow dashed line (Figure S8, Supporting Information) indicates the central axis of the main channel, along which parameters such as pressure and velocity were extracted for convergence testing. For the mesh independence test, three mesh densities were generated by progressively refining the maximum element size by ≈16%, resulting in 402892 (normal), 468076 (fine), and 543052 (finer) elements. The monitored parameters included the local pressure and velocity at a representative point on the channel axis (300, −925, 125), as well as the pressure distribution along the same axis. The relative deviation between two successive meshes was calculated as |X_1_–X_2_|/X_1_ × 100%, where X represents the monitored variable, and a deviation below 5% was taken as an indicator of mesh independence. Similarly, the time‐step independence test was conducted with time steps of 0.001 s, 0.0005 s, and 0.0001 s. The relative deviations in both pressure and velocity were within 5%, confirming the independence of the results. Therefore, the normal mesh and a time step of 0.001 s were adopted for all subsequent simulations to ensure numerical stability and computational efficiency.

### Flow Field Characterization

The flow field was characterized using a high‐speed micro‐particle image velocimetry(µPIV) system, which was established in the laboratory of Huang, as previously describe.^[^ ^56^ ^]^ The system consists of a continuous‐wave laser (MGL‐III‐532‐200 mW) for particle excitation, an Olympus LMPLANFLN 50× objective lens for imaging, and a Photron Nova S12 high‐speed camera for flow field acquisition. To visualize the flow patterns, fluorescent polystyrene particles with a diameter of 1 µm (Thermo Scientific Fluoro‐Max R0100) were dispersed in a calcium chloride aqueous solution at a volumetric ratio of 1:50. During the experiments, the CaCl_2_ solution was injected into the capillary at a constant flow rate of 1 µL min^−1^, and the same solution was supplied to the aqueous flow channel at a steady rate of 10 µL min^−1^. The flow rate of paraffin oil was systematically varied at 20, 40, and 60 µL min^−1^ to investigate the interfacial dynamics under different conditions. Prior to image acquisition, the system was carefully purged to eliminate air bubbles and ensure flow stability. Imaging parameters, including frame rate and exposure time, were adjusted for each condition to enable accurate velocity vector extraction. The CaCl_2_ droplet flows were recorded using a high‐speed camera, and the image sequences were acquired and initially processed using Dynamic Studio software (Dantec Dynamics) to extract particle displacement information. The µPIV velocity fields were then post‐processed and visualized using Tecplot 360 (Tecplot Inc.), producing color‐coded vector maps of the flow. For quantitative comparison with numerical simulations, three characteristic time points during a single droplet generation cycle were selected, and the corresponding time‐averaged velocity fields within the CaCl_2_ droplets were computed. These experimental velocity maps were then compared with the simulation results to validate the flow patterns and assess the accuracy of the numerical model.

### Cell Culture

As detailed in our previous study,^[^ ^57^ ^]^ human umbilical vein endothelial cells (HUVECs) were isolated from newborn umbilical cords. Informed consent was obtained from each donor, and all protocols involving human samples were approved by the Bioethics Committee of the School of Biological and Medical Engineering, Beihang University (Approval No. BM20220019). The primary HUVECs were cultured in endothelial cell medium (ECM), supplemented with 5% fetal bovine serum (FBS), 1% endothelial cell growth supplement (ECGS), and 1% penicillin/streptomycin solution. The cells were maintained at 37 °C in a 5% CO_2_ incubator, using passages 2–7 for the experiments.

### Cell Viability Assay

Cell viability was evaluated using the Live/Dead Viability/Cytotoxicity Kit. Cell‐laden microfibers were incubated at 37 °C for 30 min in a solution containing 2 µm calcein AM and 4 µm ethidium homodimer‐1 in phosphate‐buffered saline (PBS). Calcein AM is converted into fluorescent calcein by intracellular esterases, resulting in green fluorescence in live cells. Ethidium homodimer‐1 binds to nucleic acids in cells with compromised membrane integrity, producing red fluorescence in dead cells. Following incubation, samples were washed three times with PBS to remove excess dye. Fluorescence images were acquired using a Leica SP8 confocal fluorescence microscope (Leica Microsystems, Wetzlar, Germany). The results were analyzed to determine the proportions of live (green) and dead (red) cells.

### Statistical Analysis

All quantitative data are presented as mean ± standard deviation (SD). Sample sizes (n) are indicated in the figure legends. Data were checked for outliers and normalized when appropriate. For comparisons of more than two groups, one‐way ANOVA with Tukey's post‐hoc test was used. All tests were two‐sided, and *p* ≤ 0.05 was considered statistically significant. Analyses were performed using GraphPad Prism 8.0 (GraphPad Software, San Diego, CA, USA). Figure legends specify sample size, data presentation, statistical test, post‐hoc method, and the meaning of significance symbols (^*^ *p* < 0.05, ^**^ *p* < 0.01, ^***^ *p* < 0.001, ^****^ *p* < 0.0001).

## Conflict of Interest

The authors declare no conflict of interest.

## Author Contributions

D.Z. designed and performed the experiments, analyzed the data, and wrote the manuscript. J.L. conducted the liquid bridge experiments. H.Y., S.L., and Y.H. carried out the µPIV measurements. Y.S. optimized the design of the microfluidic chips. S.Y. assisted in the numerical simulation of two‐phase flow. H.S., Z.L., K.L., X.Z., Y.L., and Z.Z. assisted in the fabrication of microfluidic chips and cell culture. X.L. conceived the study, analyzed the data, and co‐wrote the manuscript. Y.F. directed the research, including experiment design, data analysis, and revised the manuscript. All authors approved the final version of the manuscript.

## Supporting information



Supporting Information

Supplemental Movie 1

Supplemental Movie 2

Supplemental Movie 3

Supplemental Movie 4

Supplemental Movie 5

Supplemental Movie 6

Supplemental Movie 7

Supplemental Movie 8

## Data Availability

The data that support the findings of this study are available from the corresponding author upon reasonable request.

## References

[1] H. Lee , J. Kim , H. Kim , J. Kim , S. Kwon , Nature Mater 2010, 9, 745. 20729849 10.1038/nmat2815

[2] E. Mohagheghian , J. Luo , F. M. Yavitt , F. Wei , P. Bhala , K. Amar , F. Rashid , Y. Wang , X. Liu , C. Ji , J. Chen , D. P. Arnold , Z. Liu , K. S. Anseth , N. Wang , Sci Robot 2023, 8, adc9800. 10.1126/scirobotics.adc9800 PMC10098875 36696474

[3] L. Xuan , Y. Hou , L.u Liang , J. Wu , K. Fan , L. Lian , J. Qiu , Y. Miao , H. Ravanbakhsh , M. Xu , G. Tang , Nano‐Micro Lett. 2024, 16, 218. 10.1007/s40820-024-01421-5 PMC11183039 38884868

[4] D. An , A. Chiu , J. A. Flanders , W. Song , D. Shou , Y.‐C. Lu , L. G. Grunnet , L. Winkel , C. Ingvorsen , N. S. Christophersen , J. J. Fels , F. W. Sand , Y. Ji , L. Qi , Y. Pardo , D. Luo , M. Silberstein , J. Fan , M. Ma , Proc. Natl. Acad. Sci. USA 2018, 115, E263. 29279393 10.1073/pnas.1708806115 PMC5777032

[5] C. Fernández‐Rico , M. Chiappini , T. Yanagishima , H. de Sousa , D. G. A. L. Aarts , M. Dijkstra , R. P. A. Dullens , Science 2020, 369, 950. 32820121 10.1126/science.abb4536

[6] A. T. Liu , M. Hempel , J. F. Yang , A. M. Brooks , A. Pervan , V. B. Koman , G. Zhang , D. Kozawa , S. Yang , D. I. Goldman , M. Z. Miskin , A. W. Richa , D. Randall , T. D. Murphey , T. Palacios , M. S. Strano , Nat. Mater. 2023, 22, 1453. 37620646 10.1038/s41563-023-01589-y

[7] K. O. Rojek , M. Cwiklinska , J. Kuczak , J. Guzowski , Chem. Rev. 2022, 122, 16839. 36108106 10.1021/acs.chemrev.1c00798 PMC9706502

[8] S. Choi , K. Y. Lee , S. L. Kim , L. A. MacQueen , H. Chang , J. F. Zimmerman , Q. Jin , M. M. Peters , H. A. M. Ardoña , X. Liu , A.‐C. Heiler , R. Gabardi , C. Richardson , W. T. Pu , A. R. Bausch , K. K. Parker , Nat. Mater. 2023, 22, 1039. 37500957 10.1038/s41563-023-01611-3 PMC10686196

[9] B. Kessel , M. Lee , A. Bonato , Y. Tinguely , E. Tosoratti , M. Zenobi‐Wong , Adv. Sci. 2020, 7, 2001419. 10.1002/advs.202001419 PMC7509724 32999847

[10] D. Rommel , M. Mork , S. Vedaraman , C. Bastard , L. P. B. Guerzoni , Y. Kittel , R. Vinokur , N. Born , T. Haraszti , L. De Laporte , Adv. Sci. 2022, 9, 2103554. 10.1002/advs.202103554 PMC8981485 35032119

[11] T. H. Qazi , J. Wu , V. G. Muir , S. Weintraub , S. E. Gullbrand , D. Lee , D. Issadore , J. A. Burdick , Adv. Mater. 2022, 34, 2109194. 10.1002/adma.202109194 PMC8957565 34932833

[12] J. C. Rose , M. Cámara‐Torres , K. Rahimi , J. Köhler , M. Möller , L. De Laporte , Nano Lett. 2017, 17, 3782. 28326790 10.1021/acs.nanolett.7b01123 PMC5537692

[13] J. Li , Y. Wang , L. Cai , L. Shang , Y. Zhao , Chem. Eng. J. 2022, 427, 130750.

[14] L. V. Le , P. Mohindra , Q. Fang , R. E. Sievers , M. A. Mkrtschjan , C. Solis , C. W. Safranek , B. Russell , R. J. Lee , T. A. Desai , Biomaterials 2018, 169, 11. 29631164 10.1016/j.biomaterials.2018.03.042 PMC5931400

[15] T. Thorsen , Phys. Rev. Lett. 2001, 86, 4163. 11328121 10.1103/PhysRevLett.86.4163

[16] A. M. Gañán‐Calvo , Phys. Rev. Lett. 2001, 87, 274501. 11800883 10.1103/PhysRevLett.87.274501

[17] P. Umbanhowar , V. Prasad , D. Weitz , Langmuir 2000, 16, 347.

[18] A. S. Utada , E. Lorenceau , D. R. Link , P. D. Kaplan , H. A. Stone , D. A. Weitz , Science 2005, 308, 537. 15845850 10.1126/science.1109164

[19] J. J. Kaufman , G. Tao , S. Shabahang , E.‐H. Banaei , D. S. Deng , X. Liang , S. G. Johnson , Y. Fink , A. F. Abouraddy , Nature 2012, 487, 463. 22810590 10.1038/nature11215

[20] J. Eggers , E. Villermaux , Rep. Prog. Phys. 2008, 71, 036601.

[21] D. Dendukuri , D. C. Pregibon , J. Collins , T. A. Hatton , P. S. Doyle , Nature Mater 2006, 5, 365. 16604080 10.1038/nmat1617

[22] A. Perazzo , J. K. Nunes , S. Guido , H. A. Stone , Proc. Natl. Acad. Sci. USA 2017, 114, E8557. 28923973 10.1073/pnas.1710927114 PMC5642717

[23] M. de Menech , P. Garstecki , F. Jousse , H. A. Stone , J. Fluid Mech. 2008, 595, 141.

[24] J. K. Nunes , S. S. H. Tsai , J. Wan , H. A. Stone , J. Phys. D: Appl. Phys. 2013, 46, 114002. 23626378 10.1088/0022-3727/46/11/114002 PMC3634598

[25] P. Garstecki , M. J. Fuerstman , H. A. Stone , G. M. Whitesides , Lab Chip 2006, 6, 437. 16511628 10.1039/b510841a

[26] A. Gupta , R. Kumar , Microfluid. Nanofluid. 2010, 8, 799.

[27] G. F. Christopher , N. N. Noharuddin , J. A. Taylor , S. L. Anna , Phys. Rev. E 2008, 78, 36317. 10.1103/PhysRevE.78.036317 18851153

[28] X. Zhang , R. S. Padgett , O. A. Basaran , Journal of Fluid Mechanics 1996, 329, 207.

[29] G. F. Christopher , N. N. Noharuddin , J. A. Taylor , S. L. Anna , Phys. Rev. E 2008, 78, 036317. 10.1103/PhysRevE.78.036317 18851153

[30] H. Song , M. R. Bringer , J. D. Tice , C. J. Gerdts , R. F. Ismagilov , Appl. Phys. Lett. 2003, 83, 4664. 17940580 10.1063/1.1630378 PMC2025702

[31] Z. Liu , Y. Ma , X. Wang , Y. Pang , Y. Ren , D. Li , Experimental Thermal and Fluid Science 2022, 139, 110739.

[32] D. Zhang , J. Liu , X. Liu , Y. Fan , Appl. Phys. Rev. 2025, 12, 11332.

[33] C. Yang , W. Li , Y. Zhao , L. Shang , Proc. Natl. Acad. Sci. USA 2024, 121, 2402331121. 10.1073/pnas.2402331121 PMC11252946 38959044

[34] K. A. Gopinathan , A. Mishra , B. R. Mutlu , J. F. Edd , M. Toner , Nature 2023, 622, 735. 37880436 10.1038/s41586-023-06517-3 PMC10600001

[35] J. A. Weaver , J. Melin , D. Stark , S. R. Quake , M. A. Horowitz , Nature Phys 2010, 6, 218.

[36] D. J. Preston , P. Rothemund , H. J. Jiang , M. P. Nemitz , J. Rawson , Z. Suo , G. M. Whitesides , Proc. Natl. Acad. Sci. USA 2019, 116, 7750. 30923120 10.1073/pnas.1820672116 PMC6475414

[37] C. Yang , X. Liu , X. Song , L.i Zhang , Lab Chip 2024, 24, 4514. 39206574 10.1039/d4lc00566j

[38] M. D. Neto , M. B. Oliveira , J. F. Mano , Trends Biotechnol. 2019, 37, 1011. 30902347 10.1016/j.tibtech.2019.02.008

[39] J. Zeng , Y. Zhang , Y. Gao , M. Jia , Y. Guo , X. Li , Y. Wang , C. Zhao , J. Qiu , S. McGinty , W. Miao , G. Wang , Y. Wang , ACS Nano 2025, 19, 22968. 40534137 10.1021/acsnano.5c02492

[40] P. Ghaderinejad , N. Najmoddin , Z. Bagher , M. Saeed , S. Karimi , S. Simorgh , M. Pezeshki‐Modaress , Chem. Eng. J. 2021, 420, 130465.

[41] Y. Wang , X. Hu , R. K. Kankala , D.‐Y. Yang , K. Zhu , S.‐B. Wang , Y. S. Zhang , A.‐Z. Chen , Biofabrication 2019, 12, 015011. 31553962 10.1088/1758-5090/ab47eb

[42] S. Jain , S. J. Rao , S. Mandal , C. Tropea , S. Basu , arXiv preprint arXiv:2505 2025, 18528.

[43] O. Bonhomme , J. Leng , A. Colin , Soft Matter 2012, 8, 10641.

[44] M. P. Borthakur , G. Biswas , D. Bandyopadhyay , Phys. Rev. E 2017, 96, 13115. 10.1103/PhysRevE.96.013115 29347101

[45] A. Martínez‐Calvo , M. Rubio‐Rubio , A. Sevilla , J. Fluid Mech. 2018, 834, 335.

[46] M. de Menech , P. Garstecki , F. Jousse , H. A. Stone , J. Fluid Mech. 2008, 595, 141.

[47] Y. Yu , L. Shang , J. Guo , J. Wang , Y. Zhao , Nat. Protoc. 2018, 13, 2557. 30353174 10.1038/s41596-018-0051-4

[48] M. Filippi , O. Yasa , R. D. Kamm , R. Raman , R. K. Katzschmann , Proc. Natl. Acad. Sci. USA 2022, 119, 2200741119. 10.1073/pnas.2200741119 PMC9436346 36001689

[49] Y. Zhang , C. M. J. Tan , C. N. Toepfer , X. Lu , H. Bayley , Science 2024, 386, 1024. 39607936 10.1126/science.adr0428

[50] C. Xu , Z. Zhang , J. Fu , Y. Huang , Langmuir 2017, 33, 5037. 28457137 10.1021/acs.langmuir.7b00874

[51] Z. Li , C. Ruan , X. Niu , Medicine in Novel Technology and Devices 2023, 17, 100211. 10.1016/j.medntd.2022.100205 PMC9995276 36909661

[52] Materials Today Bio 2024, 24, 100930. 10.1016/j.mtbio.2023.100930 PMC10825055 38293631

[53] M. Liu , B. Jin , M. Liu , Adv. Funct. Materials 2024, 34, 2404803.

[54] D. Loessner , C. Meinert , E. Kaemmerer , L. C. Martine , K. Yue , P. A. Levett , T. J. Klein , F. P. W. Melchels , A. Khademhosseini , D. W. Hutmacher , Nat. Protoc. 2016, 11, 727. 26985572 10.1038/nprot.2016.037

[55] M. Nakamoto , Y. Kasai , T. Tanaka , T. Yamamoto , Colloids Surf. A 2020, 603, 125250.

[56] Y. Huang , H. Ye , S. Yin , R. Gao , Z. Tao , T. Li , H. Li , Phys. Fluids 2025, 37, 032016.

[57] H. Su , T. Ma , X. Liu , L.i Wang , F. Shu , Z. Liang , D. Zhang , X. Zhang , K. Li , M. Wang , C. Xin , Y.u Zhang , J. Zhang , Y. Du , Y. Fan , Appl. Phys. Rev. 2024, 11, 011404.

