# Supplementary material for: A Microfluidic Digital Shutter of Liquid–Liquid Interface for Fabrication of Multifaceted Hydrogel Microfiber Structure
Source: Adv Sci (Weinh). 2025 Dec 29;13(10):e10989. doi: 10.1002/advs.202510989 (PMC12915157; doi:10.1002/advs.202510989)
Supplement: Supplementary file 1 — Supporting Information [file ADVS-13-e10989-s007.docx]

Supplementary information

## **A Microfluidic Digital Shutter of Liquid–Liquid Interface for Fabrication of Multifaceted Hydrogel Microfiber Structure**

*Dongrui Zhang, Jiangyue Liu, Hao Ye, Yu Shen, Haoran Su, Zhuqing Liang, Kexin Li, Xing Zhang, Shiyi Yang, Yunfei Lu, Zhexi Zhang, Sihang Liu, Yi Huang, Xiao Liu^*^ and Yubo Fan^*^*

**This PDF file includes:**

Figs. S1 to S18

Legend for movie S1-S8

**Other Supplementary Materials for this manuscript include the following:**

Movie S1 to Movies S8

## **Supplementary Figures:**
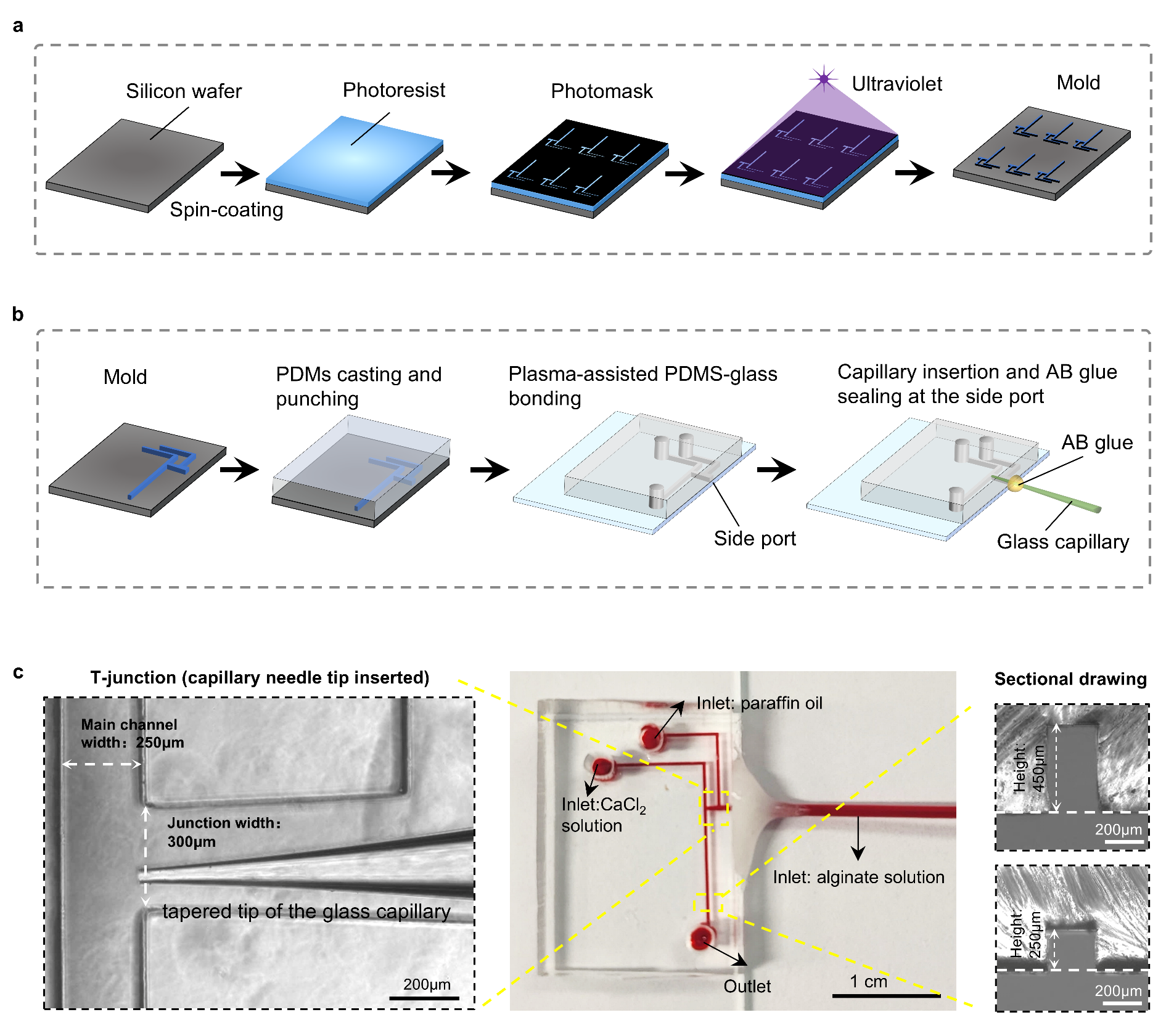


**Supplementary Figure S1 | Microfluidic setup for multifaceted microfiber production. a.** Schematic illustration of the soft-lithography process for fabricating the T-junction microfluidic chip. **b.** Assembly schematic showing the integration of the T-junction microfluidic chip with a tapered glass capillary. **c.** Schematic and microscopic views of the microfluidic chip and capillary alignment. Left: close-up image of the tapered round glass capillary inserted into the rectangular outlet channel. Middle: top-view of the PDMS microchannel system with three inlets for paraffin oil, CaCl_2_ solution, and sodium alginate solution. Right: Cross-sectional representations of the main channel, highlighting two channel heights (250 μm and 450 μm).


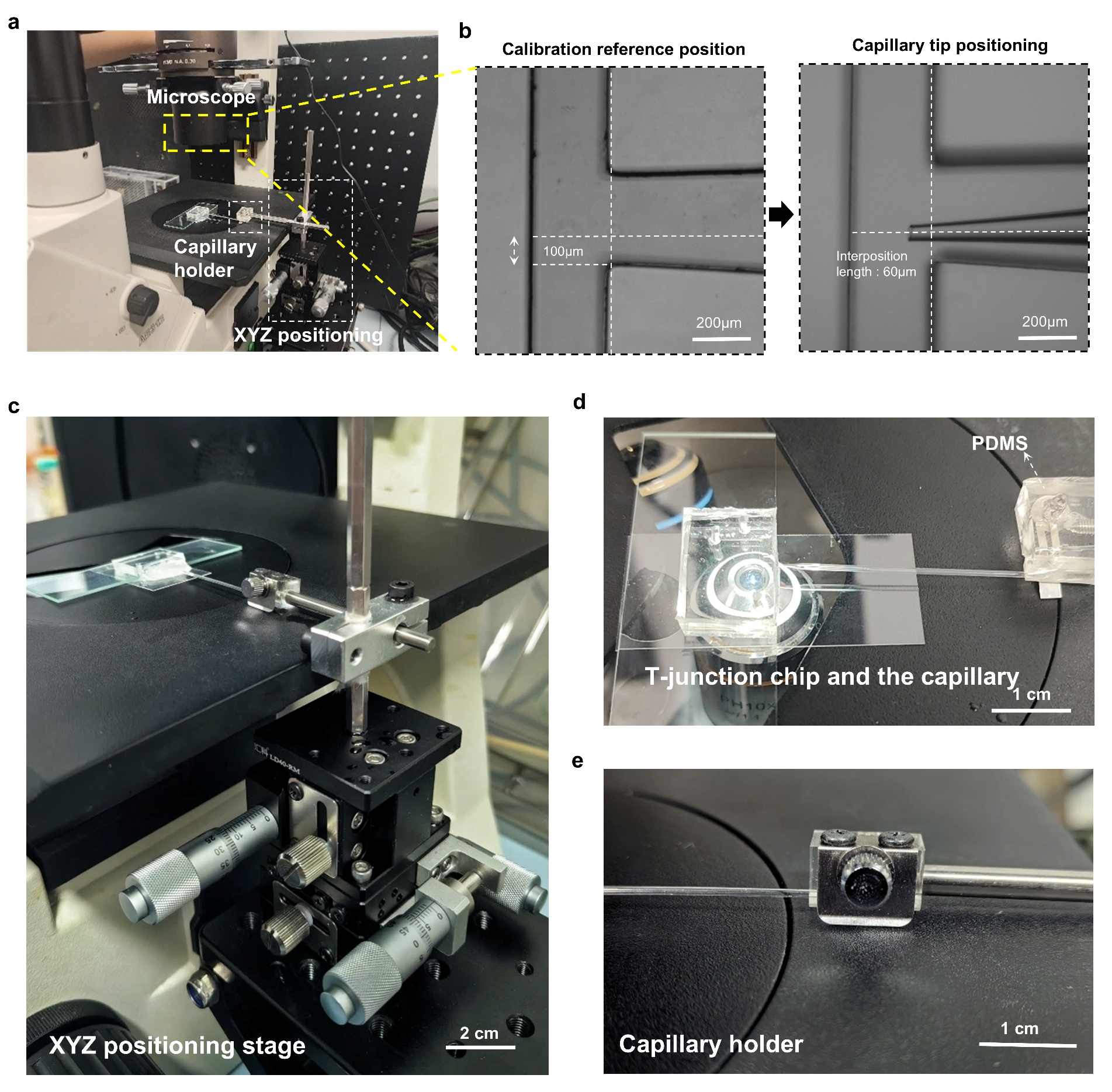


**Supplementary Figure S2 | Capillary tip positioning setup.** **a.** Capillary positioning procedure conducted under a microscope using an XYZ positioning stage and a capillary holder. **b.** Under microscopic observation, the T-junction region is imaged and aligned to a reference point. The glass capillary is carefully inserted and fixed using the capillary holder according to the insertion depth and reference alignment. **c.** Photograph of the XYZ positioning stage used for fine adjustment. **d.** Photograph showing the assembled T-junction microfluidic chip and inserted glass capillary. **e.** Photograph of the capillary holder used for precise manipulation and fixation.


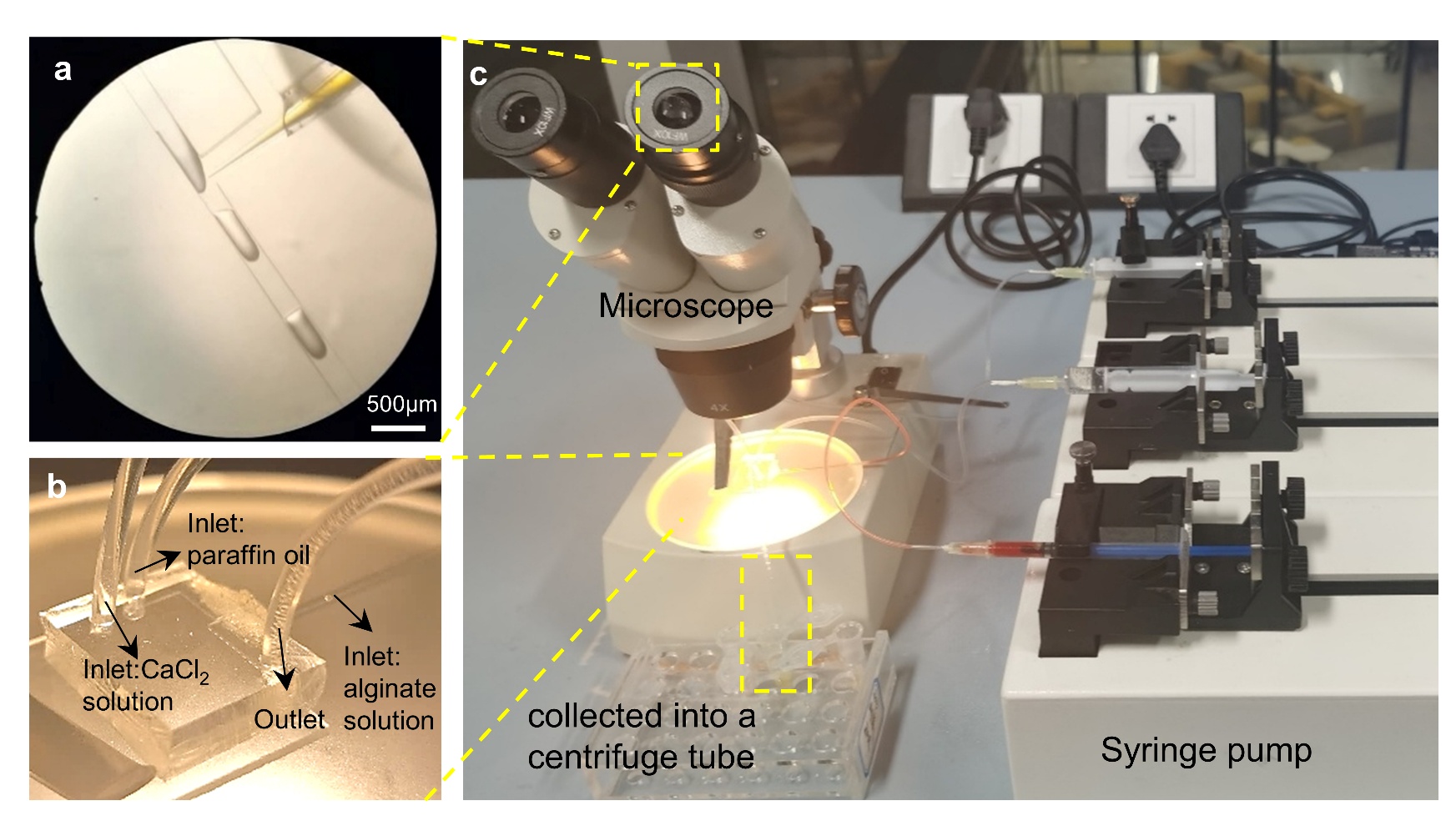


**Supplementary Figure S3 | Experimental setup for microfiber generation.** Central image **c.** stereo microscope system configured for real-time observation of droplet formation. Insets: operational view of the microchannel under the microscope (**a.** top left), and zoomed-in view of the PDMS chip showing all inlets and outlet connections (**b.** bottom left). Flow control is achieved via synchronized syringe pumps.


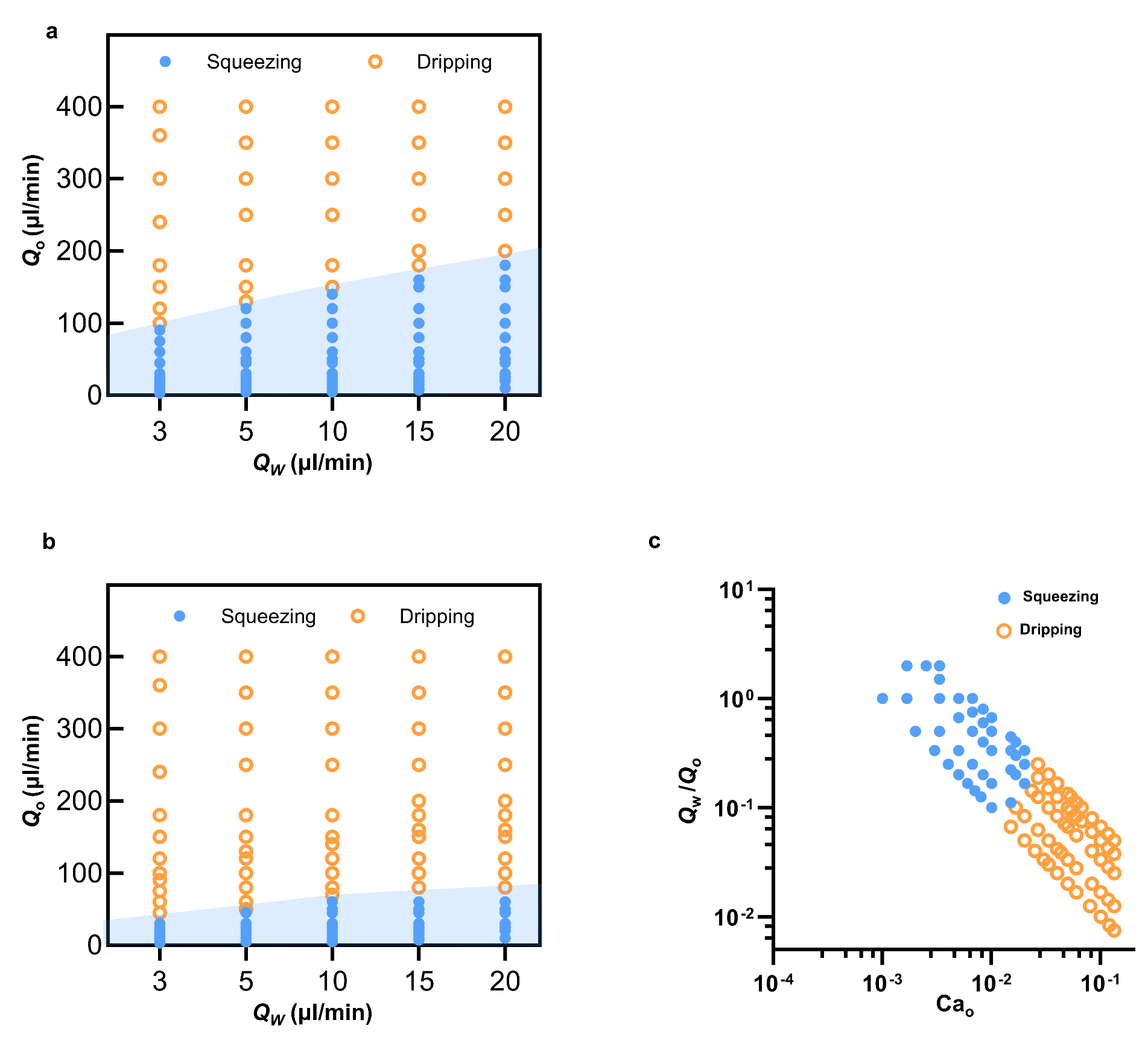


**Supplementary Figure S4 | a.** Phase diagram distinguishing squeezing and dripping regimes as a function of *Q*_w_ and *Q*_o_ in the 450 μm-height microfluidic chip. **b.** Phase diagram distinguishing squeezing and dripping regimes as a function of *Q*_w_ and *Q*_o_ in the 250 μm-height microfluidic chip. **c.** Flow regime map distinguishing the squeezing and dripping states as a function of the capillary number (Ca_o_) and the flow rate ratio (*Q*_w_*/Q*_o_) in the 250 μm-height microfluidic chip.


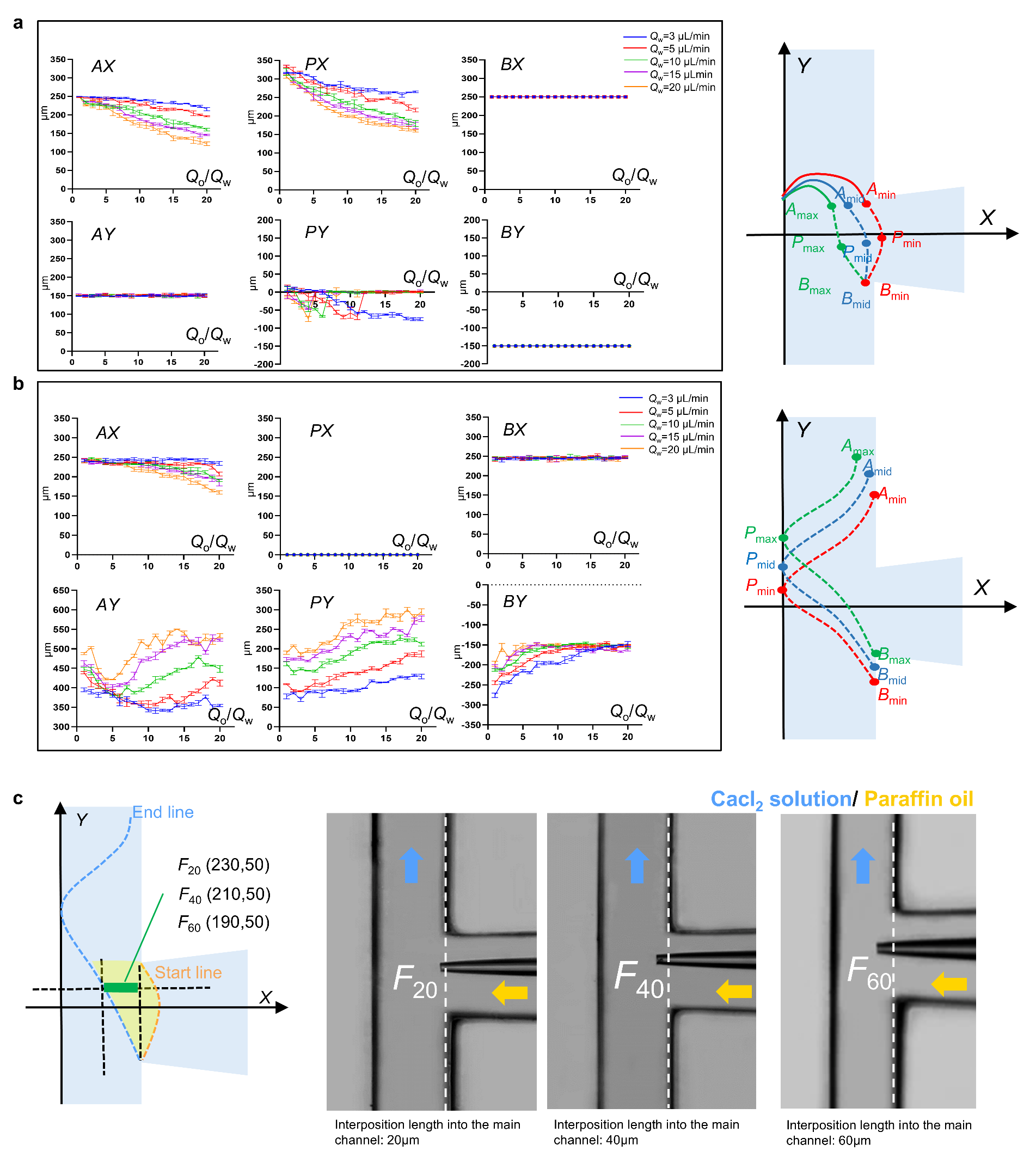


**Supplementary Figure S5 | Characterization of the interface dynamics and identification of the feasible insertion zone for capillary alignment. a.** Analysis of characteristic positions (*A*, *P*, *B*) on the liquid-liquid interface at the initial stage of CaCl_2_ droplet formation under varying flow rate ratios (*Q*_o_ / *Q*_w_). With increasing *Q*_o_ at a fixed *Q*_w_, the x-coordinate of point *A* decreases while the y-coordinate remains nearly unchanged. Both coordinates of point *P* decrease, whereas point *B* remains stable (left). This trend indicates a morphological transition of the interface from a convex to a concave parabolic profile, driven by enhanced squeezing and shear forces from the continuous phase (right). Data are presented as mean ± SD (n = 3). **b.** Characterization of the interface morphology at the end stage of CaCl_2_ droplet formation under different flow rate ratios. As flow rate ratio increases, the y-coordinates of *A*, *P*, and *B* decrease, while the x-coordinate of A shows only a slight reduction (left). At this stage, the interface consistently exhibits a concave, groove-like parabolic profile, gradually shifting downstream with increasing flow rate ratios (right). Data are presented as mean ± SD (n = 3). **c.** Feasible region for capillary tip placement (yellow), bounded by the initial and final interfaces at the lowest and highest flow rate ratios, respectively (left). Positions *F*_20_, *F*_40_, and *F*_60_ correspond to interposition length of 20, 40, and 60μm (right).


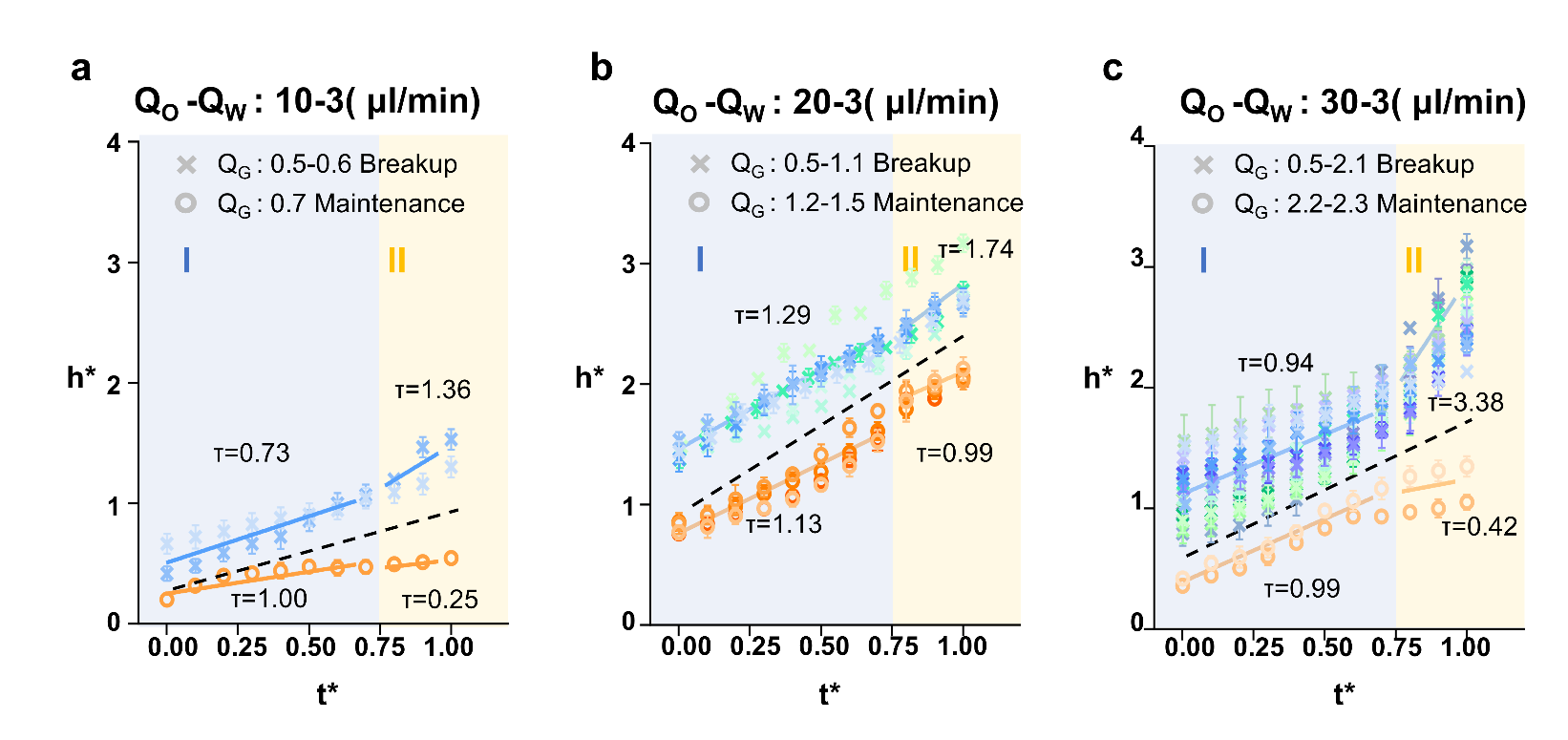


**Supplementary Figure S6 | Time evolution of the dimensionless length of the liquid bridge *h** as a function of the characteristic time *t** under different flow conditions. a.** *Q*_o_-*Q*_w_=10-3 μL/min, **b.** *Q*_o_-*Q*_w_=20-3 μL/min, **c.** *Q*_o_-*Q*_w_ = 30-3 μL/min. Solid lines represent linear fits applied to distinct regimes, with the slope *k* indicating the scaling factor of bridge growth. The fitted *k* values are annotated in the plot. Data are presented as mean ± SD (n = 3).


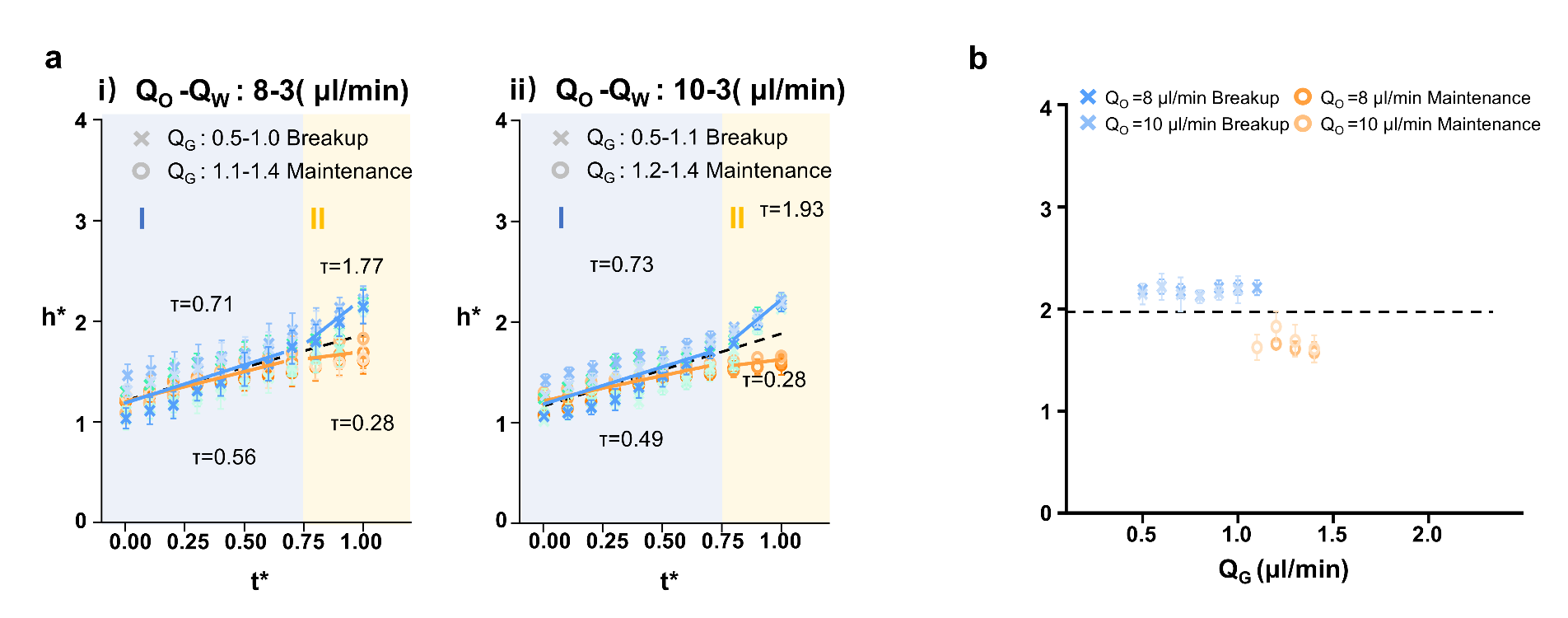


**Supplementary Figure S7 |** **The dimensionless length of the liquid bridge in a 250μm-Height Microfluidic Chip. a.** Time evolution of the dimensionless length of the liquid bridge h* as a function of the characteristic time *t** under different flow conditions: (i) *Q*_o_-*Q*_w_ = 8-3 μL/min, (ii) *Q*_o_-*Q*_w_ = 10-3 μL/min. Solid lines represent linear fits applied to distinct regimes, with the slope k indicating the scaling factor of bridge growth. The fitted k values are annotated in the plot. Data are presented as mean ± SD (n = 3). **b.** Statistical distribution of the limiting bridge length (*h**max) at *t** = 1. Data are presented as mean ± SD (n = 3).


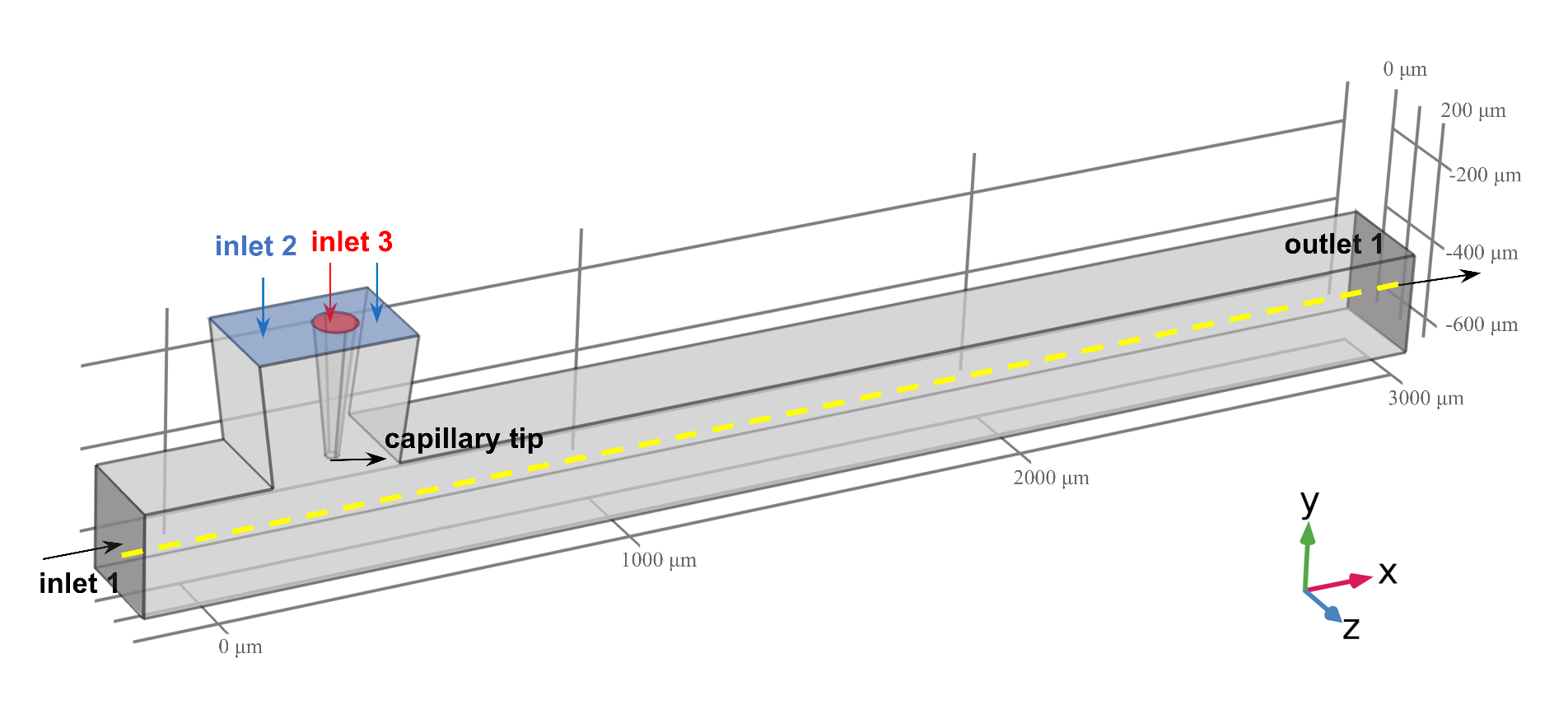


**Supplementary Figure S8 | Geometry and boundary conditions of the numerical simulation setup.** The model illustrates the two-phase flow configuration, including the inlet, outlet, and wall boundary conditions. The yellow dashed line represents the central axis of the main channel, along which parameters such as pressure, velocity were extracted for the mesh and time-step independence analyses.


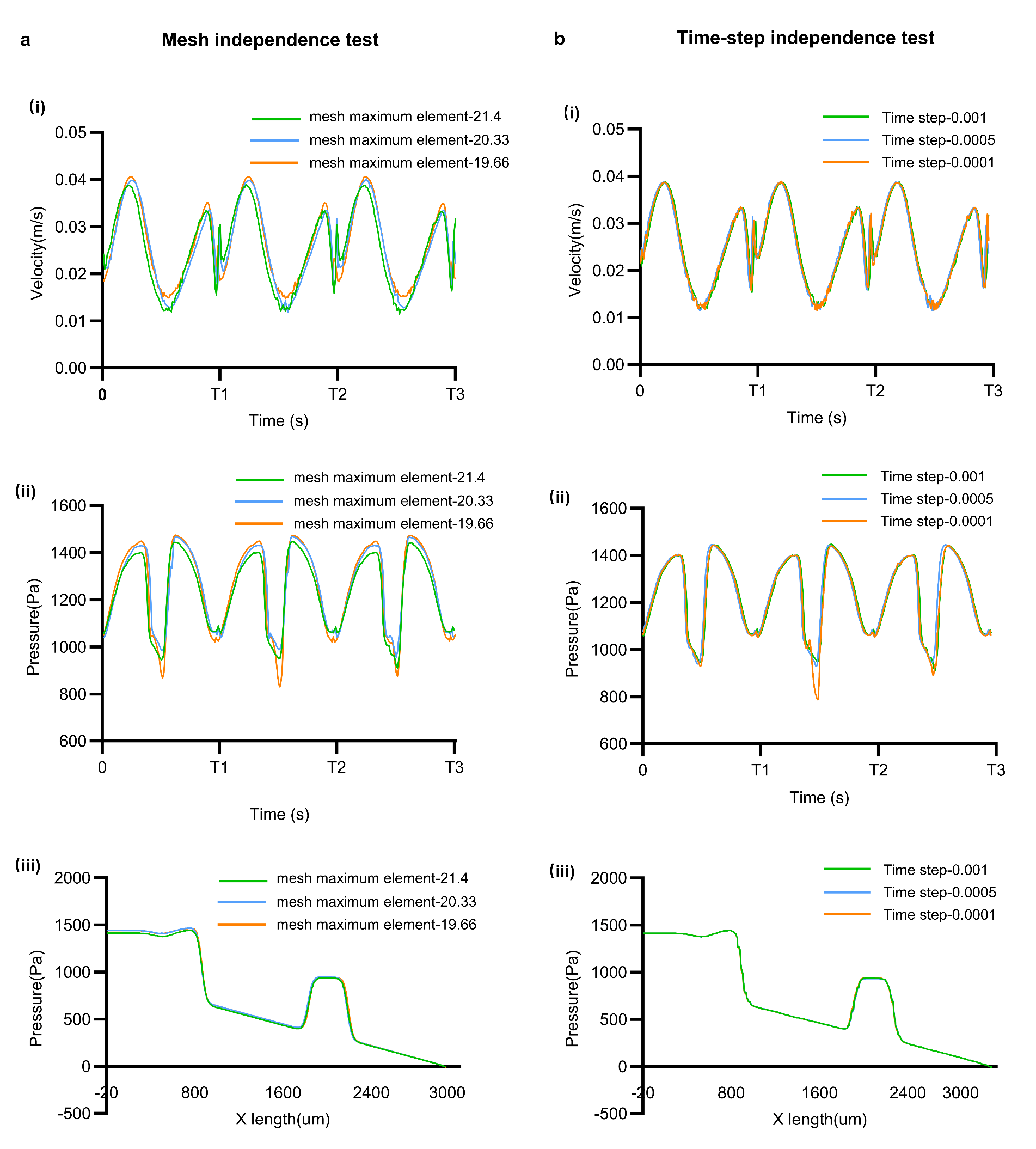


**Supplementary Figure S9 | Mesh and time-step independence analyses were conducted by comparing velocity and pressure profiles along the channel centerline (the yellow dashed line represents in Supplementary Figure S8). a.** Mesh independence test, **b.** Time-step independence test. The deviations in both parameters were within 5%, confirming that the selected normal mesh (maximum element size: 21.4 μm) and time step of 0.001s ensure stable and accurate simulation results.


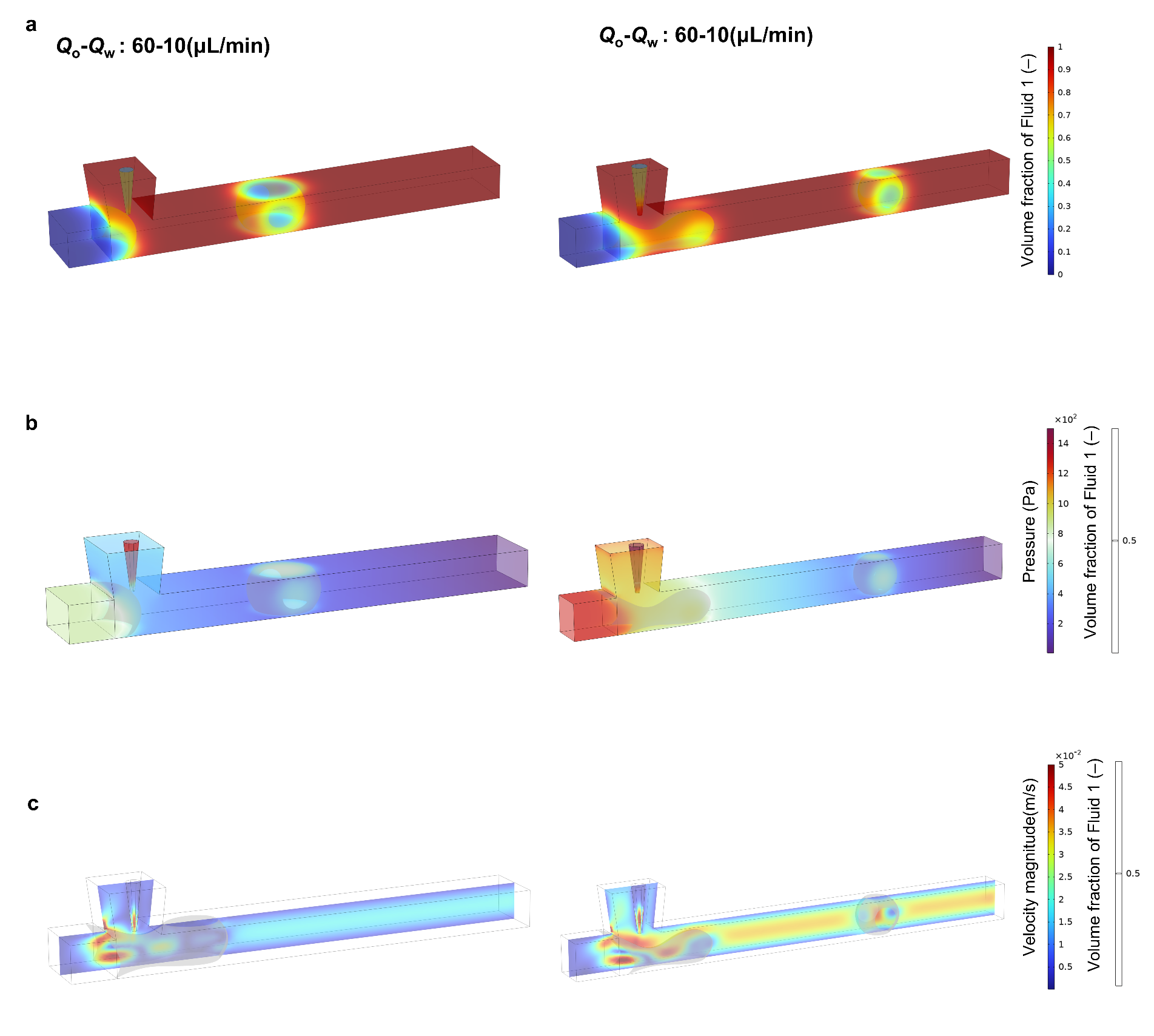


**Supplementary Figure S10 | Numerical simulation comparison of 450 μm- and 250 μm-height microfluidic chips under *Q*_G_ -*Q*_o_- *Q*_w_ : 1-60-10 μL/min. a.** Volume fraction of fluid 1 showing flow regimes in both chips. The 450 μm-height chip exhibits a squeezing regime, whereas the 250 μm-height chip exhibits a dripping regime. **b.** Pressure distribution along the channel. The 250 μm-height chip shows a higher average pressure compared to the 450 μm chip. **c.** Velocity magnitude distribution along the channel. The 250 μm-height chip exhibits a higher average velocity than the 450 μm-height chip.


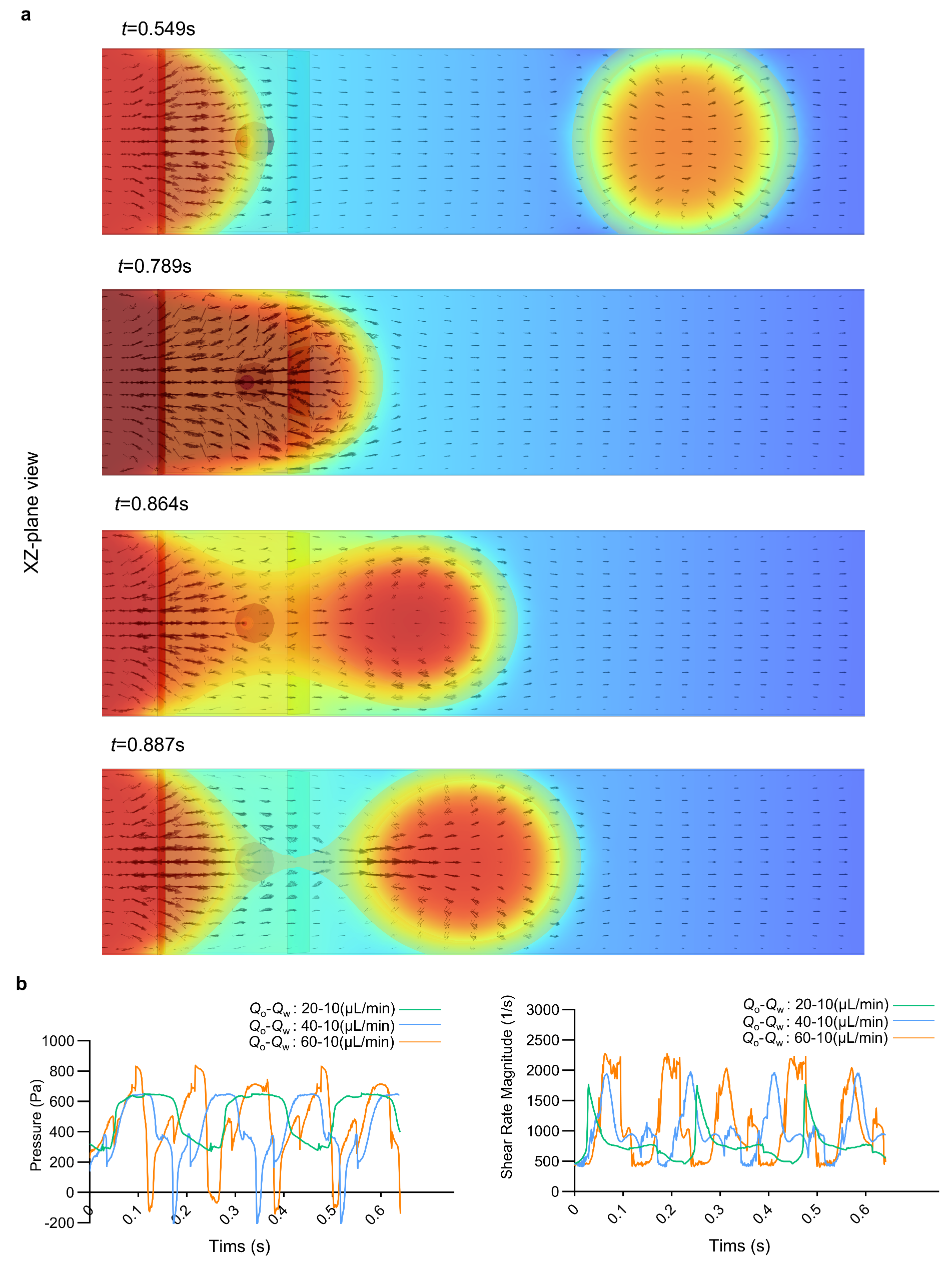


**Supplementary Figure S11 | Numerical simulation of liquid bridge dynamics. a.** 3D numerical simulation of the multiphase flow illustrating the effect of needle insertion on digital shutter formation. Black arrows represent the flow field, while the color map indicates the pressure distribution. The view shown corresponds to the x-z plane. **b.** Temporal profiles of pressure and shear rate at the capillary tip for varying oil flow rates. Both pressure and shear rate exhibit periodic fluctuations, with higher frequencies observed at increased.


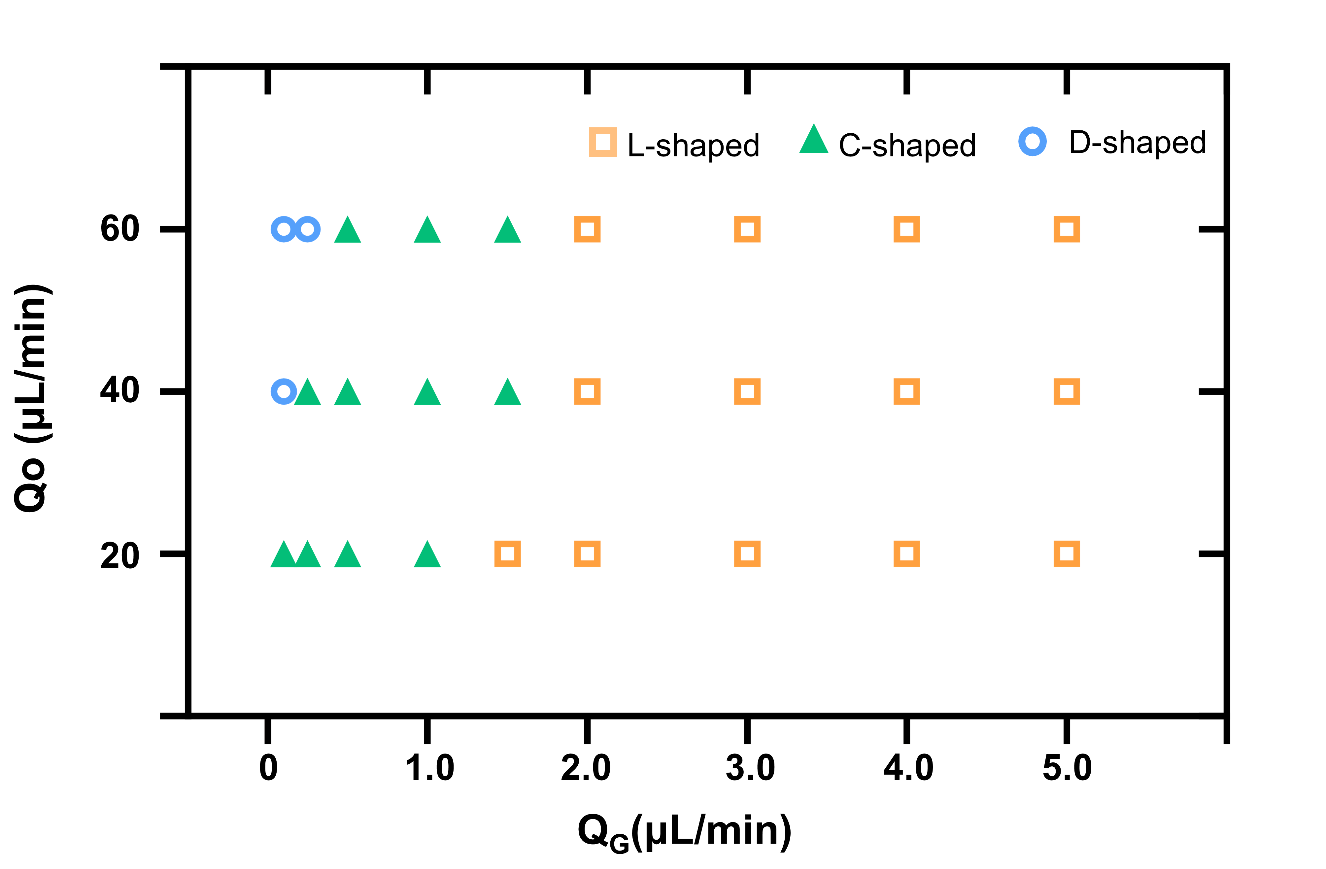


**Supplementary Figure S12 | The diagram depicts the correlation between microfiber morphology and flow conditions, including the flow rates of sodium alginate (*Q*_G_) and oil phase (*Q*_o_).** The aqueous flow rate (*Q*_w_) was fixed at 10 μL/min, with a sodium alginate concentration of 0.5% and interposition length (F) is 40 μm.


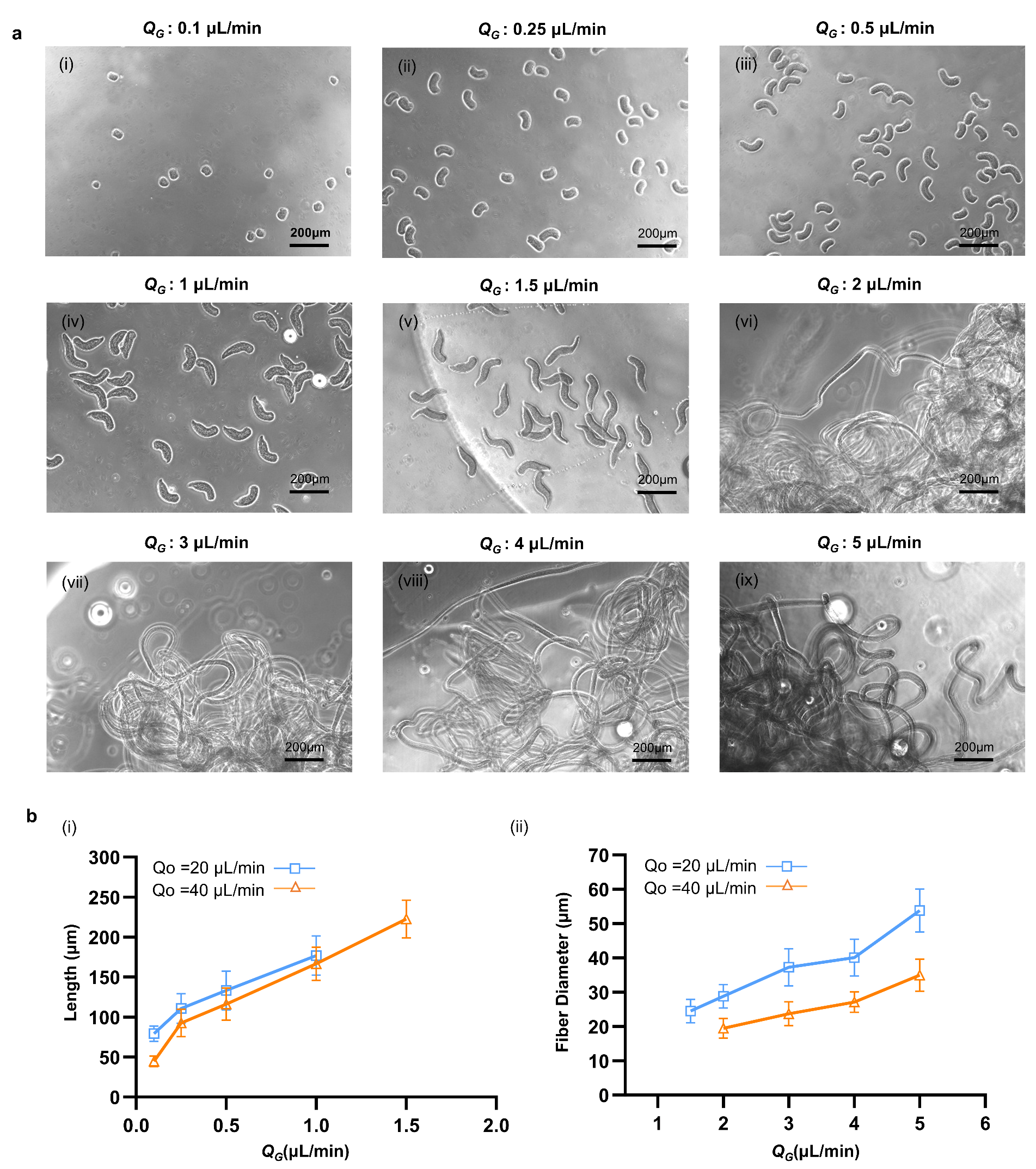


**Supplementary Figure S13 | Morphological evolution and quantitative characterization of microfibers under varying sodium alginate flow rates (*Q*_G_). a.** Representative images of microfibers exhibiting the morphological transition from D-C-L shaped as increases *Q*_G_. **b.** Quantitative analysis showing that the length of C-type microfibers increases with increasing *Q*_G_ (left), while the diameter of L-type microfibers also increases with *Q*_G_ (right). Data are presented as mean ± SD (n = 50-100).


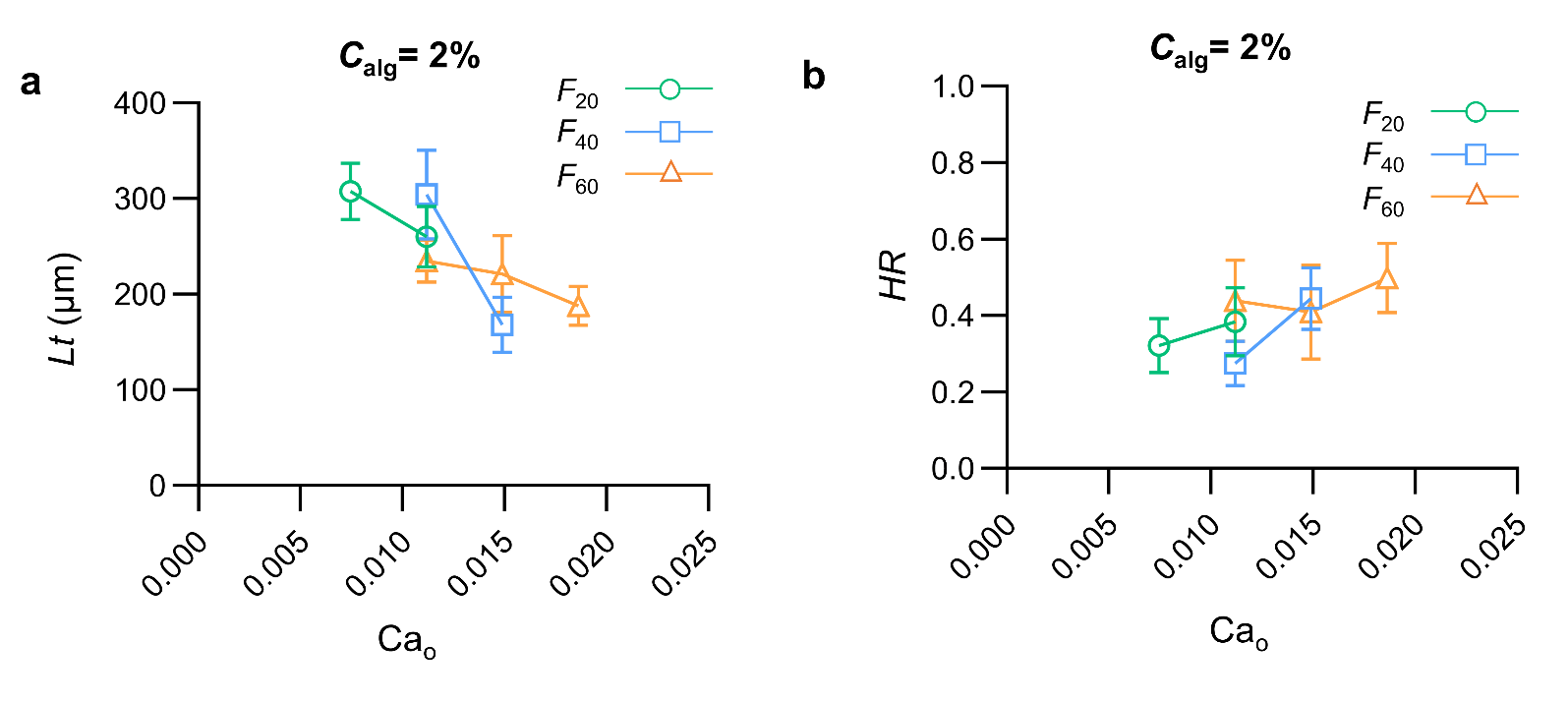


**Supplementary Figure S14 |** **Effect of 2% alginate concentration on *Lt* and *HR* as a function of Cao. a.** The total fiber length (*Lt*) as a function of Ca_o_ for alginate concentrations of 2% (w/v). Measurements were taken at different interposition length (*F*_20_, *F*_40_ and *F*_60_). Data are presented as mean± SD (n = 50-100). **b.** The head ratio (*HR*)as a function of Ca_o_ for alginate concentrations of 2% (w/v). Measurements were taken at different interposition length (*F*_20_, *F*_40_ and *F*_60_). Data are presented as mean± SD (n = 50-100).


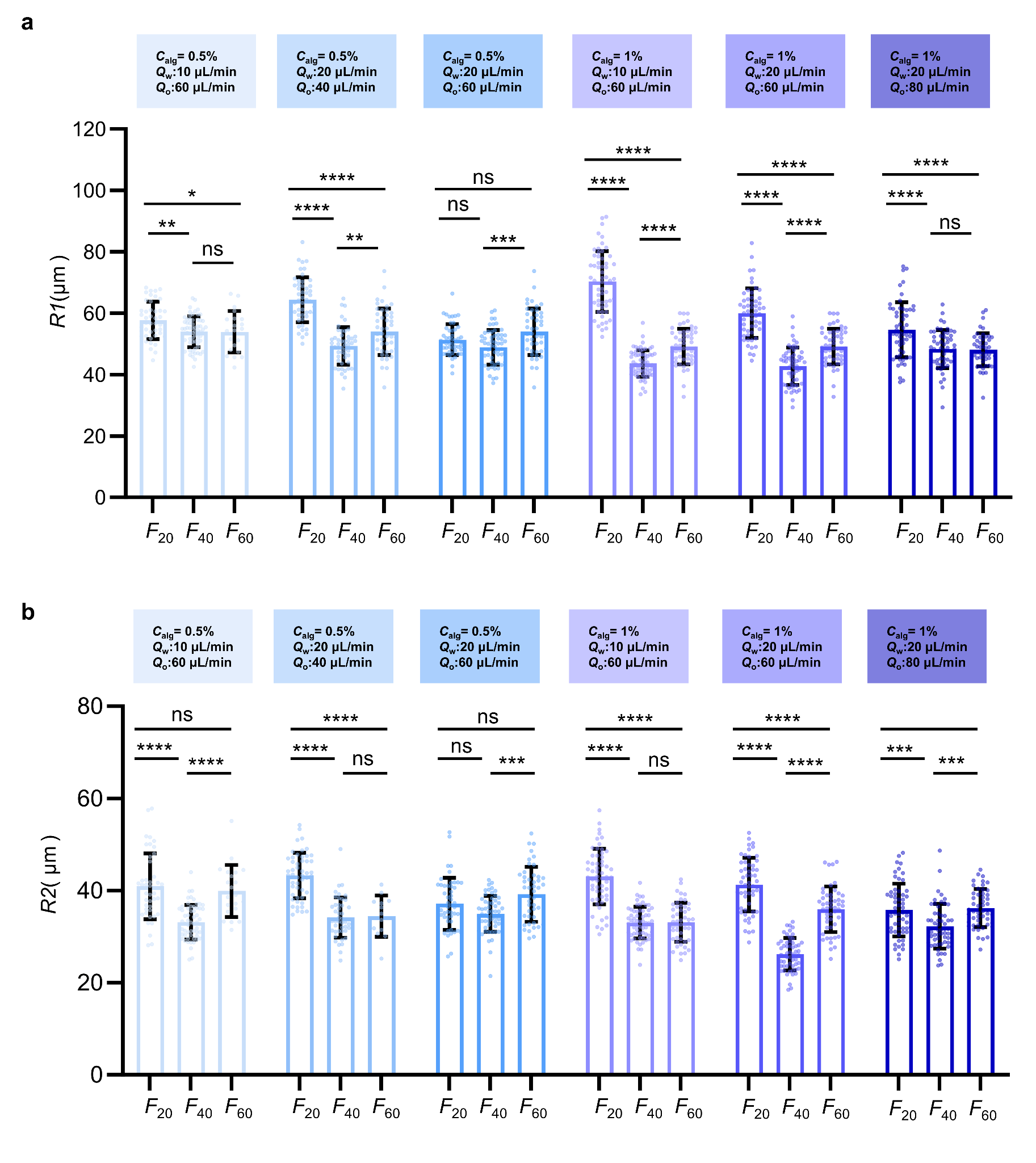


**Supplementary Figure S15 |** **Position-dependent variation in *R1* and *R2*. a.** Statistical analysis of *R1* for microfibers generated under identical conditions but at different interposition length. Data are presented as mean ± SD (n = 50-100). Statistical significance was determined by one-way ANOVA followed by Tukey’s post-hoc test (**P* < 0.05, ***P* < 0.01, ****P* < 0.001, *****P* < 0.0001; NS, not significant). **b.** Statistical analysis of *R2* for microfibers generated under identical conditions but at different interposition length. Data are presented as mean ± SD (n = 50-100). Statistical significance was determined by one-way ANOVA followed by Tukey’s post-hoc test (**P* < 0.05, ***P* < 0.01, ****P* < 0.001, *****P* < 0.0001; NS, not significant).


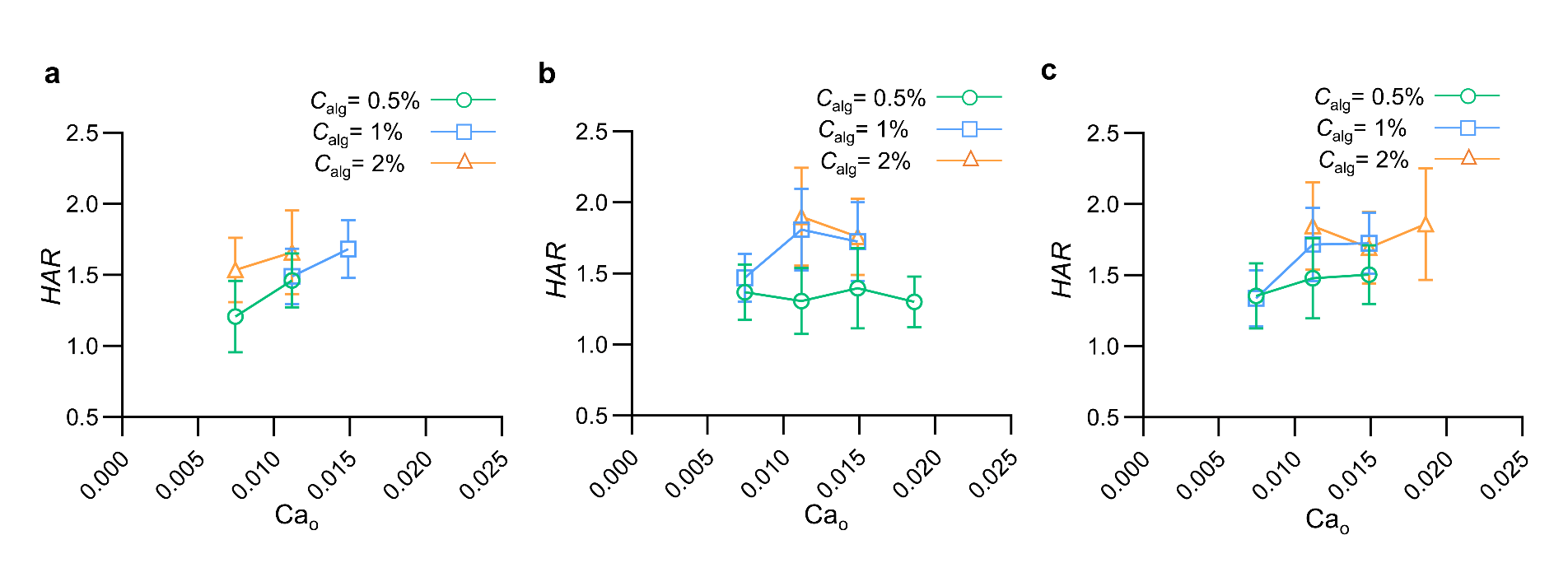


**Supplementary Figure S16 | Effect of alginate concentration on the *HAR* at different interposition length.** The head aspect ratio (*HAR*) as a function of Ca_o_ for interposition length of **a.** *F*_20_, **b.** *F*_40_, **c.** *F*_60_. Measurements were taken at different alginate concentrations (0.5%, 1%, and 2% w/v). Data are presented as mean ± SD. (n = 50-100).


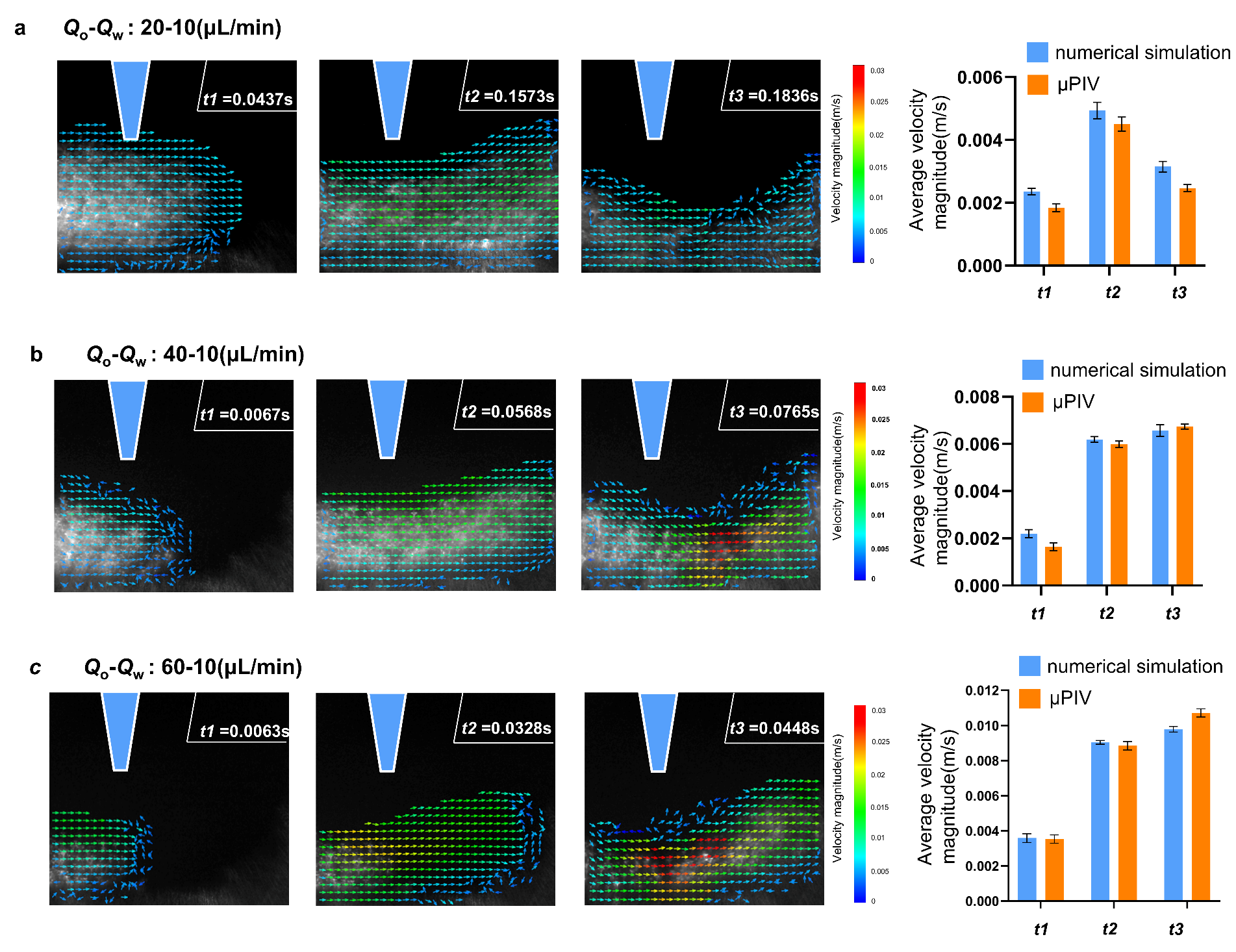


**Supplementary Figure S17 | Comparison of µPIV experimental and simulated velocity fields.** µPIV measurements showing the magnitude of internal velocity within CaCl_2_ droplets during neck contraction at different *Q*_o._ Velocity fields were measured with *Q*_w_ fixed at 10 μL/min under three *Q*_o_ conditions: **a.** 20 μL/min, **b.** 40 μL/min, and **c.** 60 μL/min. The right panel shows the comparison of average velocities obtained from μPIV measurements and numerical simulations, with deviations ranging from 5% to 20%. Experimental data are presented as mean ± SD (n = 3).


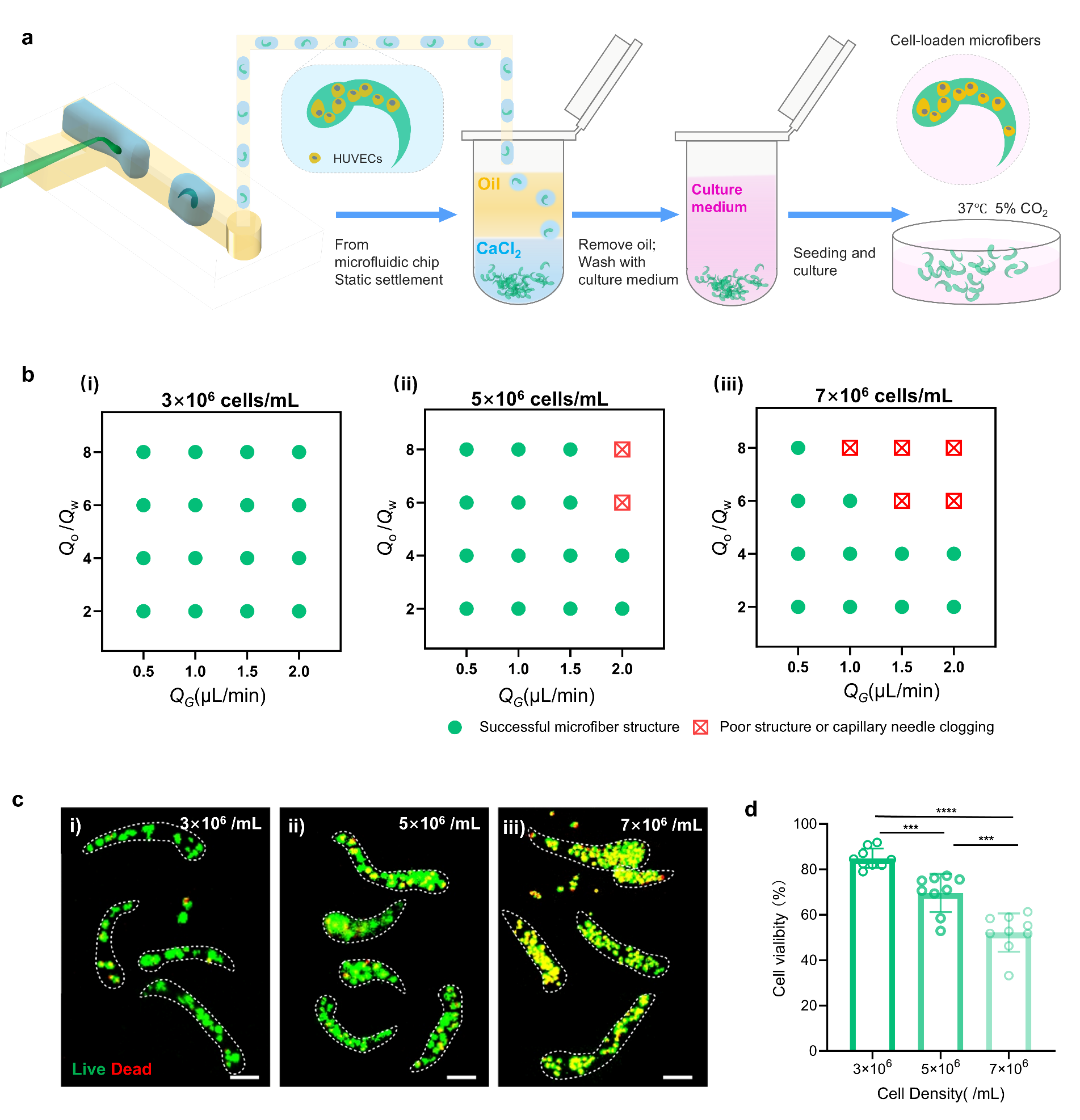


**Supplementary** **Figure S18 | a.** Schematic of the biofabrication process for endothelial cell-laden C-shaped hydrogel microfibers using our microfluidic system. **b.** Evaluation of C-type microfiber morphology at different cell densities: 3×10^6^ cells/mL, 5×10^6^ cells/mL, and 7×10^6^ cells/mL. Microfiber formation was assessed across a range of flow rate ratios (*Q*_o_ / *Q*_w_) and *Q*_G_. **c.** Confocal microscopy images illustrating cell viability within microfibers 1 day after fabrication at varying cell densities (3×10^6^, 5×10^6^, and 7×10^6^ cells/mL). Scale bar: 50 μm c. Cell viability post-encapsulation at varying cell densities. Data are presented as mean ± SD (n = 9). Statistical significance was determined by one-way ANOVA followed by Tukey’s post-hoc test, ****P* < 0.001, *****P* < 0.0001.

## **Legend for movie S1 to S8**

**Supplementary** **Movie S1 | Squeezing regime in a T-junction microfluidic device.** Real-time recording of droplet formation in the squeezing regime. Captured at *Q*_w_=10 µL/min and *Q*_o_=130 µL/min.

**Supplementary Movie S2 | Dripping regime in a T-junction microfluidic device.** Real-time recording of droplet formation in the dripping regime. Captured at *Q*_w_=10 µL/min and *Q*_o_=60 µL/min.

**Supplementary Movie S3 | Breakup of the liquid bridge following alginate–CaCl_2_ coalescence.** Real-time recording of the ductile stretching, capillary thinning, and final pinch-off of the liquid bridge. Captured at *Q*_G_=0.9 µL/min, *Q*_w_=9 µL/min and *Q*_o_=30 µL/min.

**Supplementary Movie S4 | Liquid bridge maintenance following alginate–CaCl_2_ coalescence.** Real-time recording of a liquid bridge that remains stable without rupture throughout the observation period. Captured at *Q*_G_=2.2 µL/min, *Q*_w_=9 µL/min and *Q*_o_=30 µL/min.

**Supplementary Movie S5 | Continuous gelation in the ON mode.** Real-time recording of uninterrupted gelation, resulting in the formation of elongated continuous fibers. Captured at *Q*_G_=1 µL/min, *Q*_w_=10 µL/min and *Q*_o_=20 µL/min.

**Supplementary Movie S6 | Gelation halted in the OFF mode.** Real-time recording of the cessation of gelation in the OFF state, where alginate droplets disperse in oil and flow downstream. Captured at *Q*_G_=1 µL/min, *Q*_w_=10 µL/min and *Q*_o_=100 µL/min.

**Supplementary Movie S7 | Periodic ON-OFF gelation cycles mode.** Real-time recording of the alternating ON-OFF gelation mode, regulating gelation cycles and resulting in the generation of monodisperse hydrogel structures. Captured at *Q*_G_=1 µL/min, *Q*_w_=10 µL/min and *Q*_o_=40 µL/min.

**Supplementary Movie S8 | Two-phase flow dynamics in the 450μm-height microfluidic chip.** Three-dimensional numerical simulation of dynamic two-phase flow in the 450μm-height microfluidic device at flow rates of at *Q*_G_=1 µL/min, *Q*_w_=10 µL/min and *Q*_o_=40 µL/min. The colored field inside the chip represents the pressure distribution, while the black arrows indicate the local velocity vectors. The interface between oil and water is highlighted by the iso-surface of fluid 1 volume fraction at 0.5, showing the formation and evolution of droplets within the channel.
